# Core microbiome-associated proteins associated with ulcerative colitis interact with cytokines for synergistic or antagonistic effects on gut bacteria

**DOI:** 10.1093/ismejo/wrae146

**Published:** 2024-07-29

**Authors:** Ting Zhang, Hang Zhong, Lu Lin, Zhiyan Zhang, Kewen Xue, Feixiang He, Yingshu Luo, Panpan Wang, Zhi Zhao, Li Cong, Pengfei Pang, Xiaofeng Li, Hong Shan, Zhixiang Yan

**Affiliations:** Guangdong Provincial Engineering Research Center of Molecular Imaging, The Fifth Affiliated Hospital, Sun Yat-sen University, and Southern Marine Science and Engineering Guangdong Laboratory (Zhuhai), Meihua East Road, Zhuhai, Guangdong 519000, China; Guangdong-Hong Kong-Macao University Joint Laboratory of Interventional Medicine, The Fifth Affiliated Hospital, Sun Yat-sen University, Meihua East Road, Zhuhai, Guangdong 519000, China; Guangdong Provincial Engineering Research Center of Molecular Imaging, The Fifth Affiliated Hospital, Sun Yat-sen University, and Southern Marine Science and Engineering Guangdong Laboratory (Zhuhai), Meihua East Road, Zhuhai, Guangdong 519000, China; Guangdong-Hong Kong-Macao University Joint Laboratory of Interventional Medicine, The Fifth Affiliated Hospital, Sun Yat-sen University, Meihua East Road, Zhuhai, Guangdong 519000, China; Department of Gastroenterology, The Fifth Affiliated Hospital, Sun Yat-sen University, Meihua East Road, Zhuhai, Guangdong 519000, China; Guangdong Provincial Engineering Research Center of Molecular Imaging, The Fifth Affiliated Hospital, Sun Yat-sen University, and Southern Marine Science and Engineering Guangdong Laboratory (Zhuhai), Meihua East Road, Zhuhai, Guangdong 519000, China; Guangdong-Hong Kong-Macao University Joint Laboratory of Interventional Medicine, The Fifth Affiliated Hospital, Sun Yat-sen University, Meihua East Road, Zhuhai, Guangdong 519000, China; Guangdong Provincial Engineering Research Center of Molecular Imaging, The Fifth Affiliated Hospital, Sun Yat-sen University, and Southern Marine Science and Engineering Guangdong Laboratory (Zhuhai), Meihua East Road, Zhuhai, Guangdong 519000, China; Guangdong-Hong Kong-Macao University Joint Laboratory of Interventional Medicine, The Fifth Affiliated Hospital, Sun Yat-sen University, Meihua East Road, Zhuhai, Guangdong 519000, China; Guangdong Provincial Engineering Research Center of Molecular Imaging, The Fifth Affiliated Hospital, Sun Yat-sen University, and Southern Marine Science and Engineering Guangdong Laboratory (Zhuhai), Meihua East Road, Zhuhai, Guangdong 519000, China; Guangdong-Hong Kong-Macao University Joint Laboratory of Interventional Medicine, The Fifth Affiliated Hospital, Sun Yat-sen University, Meihua East Road, Zhuhai, Guangdong 519000, China; Department of Gastroenterology, The Fifth Affiliated Hospital, Sun Yat-sen University, Meihua East Road, Zhuhai, Guangdong 519000, China; Department of Endocrinology and Metabolism, The Fifth Affiliated Hospital, Sun Yat-sen University, Meihua East Road, Zhuhai, Guangdong 519000, China; Department of Endocrinology and Metabolism, The Fifth Affiliated Hospital, Sun Yat-sen University, Meihua East Road, Zhuhai, Guangdong 519000, China; Department of Endocrinology and Metabolism, The Fifth Affiliated Hospital, Sun Yat-sen University, Meihua East Road, Zhuhai, Guangdong 519000, China; Guangdong Provincial Engineering Research Center of Molecular Imaging, The Fifth Affiliated Hospital, Sun Yat-sen University, and Southern Marine Science and Engineering Guangdong Laboratory (Zhuhai), Meihua East Road, Zhuhai, Guangdong 519000, China; Guangdong-Hong Kong-Macao University Joint Laboratory of Interventional Medicine, The Fifth Affiliated Hospital, Sun Yat-sen University, Meihua East Road, Zhuhai, Guangdong 519000, China; Department of Gastroenterology, The Fifth Affiliated Hospital, Sun Yat-sen University, Meihua East Road, Zhuhai, Guangdong 519000, China; Guangdong Provincial Engineering Research Center of Molecular Imaging, The Fifth Affiliated Hospital, Sun Yat-sen University, and Southern Marine Science and Engineering Guangdong Laboratory (Zhuhai), Meihua East Road, Zhuhai, Guangdong 519000, China; Guangdong-Hong Kong-Macao University Joint Laboratory of Interventional Medicine, The Fifth Affiliated Hospital, Sun Yat-sen University, Meihua East Road, Zhuhai, Guangdong 519000, China; Guangdong Provincial Engineering Research Center of Molecular Imaging, The Fifth Affiliated Hospital, Sun Yat-sen University, and Southern Marine Science and Engineering Guangdong Laboratory (Zhuhai), Meihua East Road, Zhuhai, Guangdong 519000, China; Guangdong-Hong Kong-Macao University Joint Laboratory of Interventional Medicine, The Fifth Affiliated Hospital, Sun Yat-sen University, Meihua East Road, Zhuhai, Guangdong 519000, China

**Keywords:** metaproteomic, inflammatory bowel disease, gut microbiome, S100a8, S100a9, cytokines

## Abstract

Inflammatory bowel disease (IBD), including Crohn’s disease (CD) and ulcerative colitis (UC), is associated with a loss or an imbalance of host–microorganism interactions. However, such interactions at protein levels remain largely unknown. Here, we applied a depletion-assisted metaproteomics approach to obtain in-depth host–microbiome association networks of IBD, where the core host proteins shifted from those maintaining mucosal homeostasis in controls to those involved in inflammation, proteolysis, and intestinal barrier in IBD. Microbial nodes such as short-chain fatty-acid producer-related host–microbial crosstalk were lost or suppressed by inflammatory proteins in IBD. Guided by protein–protein association networks, we employed proteomics and lipidomics to investigate the effects of UC-related core proteins S100A8, S100A9, and cytokines (IL-1β, IL-6, and TNF-α) on gut bacteria. These proteins suppressed purine nucleotide biosynthesis in stool-derived *in vitro* communities, which was also reduced in IBD stool samples. Single species study revealed that S100A8, S100A9, and cytokines can synergistically or antagonistically alter gut bacteria intracellular and secreted proteome, with combined S100A8 and S100A9 potently inhibiting beneficial *Bifidobacterium adolescentis*. Furthermore, these inflammatory proteins only altered the extracellular but not intracellular proteins of *Ruminococcus gnavus*. Generally, S100A8 induced more significant bacterial proteome changes than S100A9, IL-1β, IL-6, and TNF-α but gut bacteria degrade significantly more S100A8 than S100A9 in the presence of both proteins. Among the investigated species, distinct lipid alterations were only observed in *Bacteroides vulgatus* treated with combined S100A8, S100A9, and cytokines. These results provided a valuable resource of inflammatory protein-centric host–microbial molecular interactions.

## Introduction

Inflammatory bowel disease (IBD) is a persistent gastrointestinal inflammatory disease, consisting of Crohn’s disease (CD) and ulcerative colitis (UC), likely arising from the imbalanced interactions between host genetic, microbial, and environmental factors [1–6]. However, such interactions at protein levels remain largely unknown.

Metaproteomics holds the promise to promote our understanding of how gut microorganisms interact with each other and with their host [7–11], but suffers from the insufficient sensitivity to identify low-abundance microbial species and proteins. Several strategies such as immunodepletion, enrichment, and fractionation have been employed to increase the depth of proteomics [12–15] or metaproteomics [8, 9]. A recent study has revealed that abundant proteins in cells can be depleted using an ultralow concentration of trypsin predigestion [16, 17]. However, this strategy has not been evaluated in more complex biological samples such as stool.

In this study, we developed a trypsin predigestion–based deep metaproteomic approach, with which we found altered host–microbial protein association networks in IBD. Focused on core inflammation–associated proteins, our study demonstrated that S100A8 and S100A9 can act on gut bacteria synergistically or antagonistically with cytokines IL-1β and TNF-α.

## Materials and Methods

### Subjects and stool sample collections

A total of 89 subjects (26 CD patients, 29 UC patients, and 34 controls) were recruited from The Fifth Affiliated Hospital of Sun Yat-sen University (Table S1). IBD was diagnosed based on criteria regarding clinical symptoms, laboratory examination, imaging, endoscopy, and histopathology [18, 19]. Control subjects met the following criteria: no gut mucosa inflammation, no infection diseases, and no antibiotics treatment within 4 weeks. Informed consent was obtained from each subject. Stool samples were collected on ice and transferred to the laboratory to be stored at −80°C immediately. Before the sample collection, none of the patients had received any medical treatment. The severity of CD and UC was evaluated on the CDAI (Crohn’s disease activity index) [20] and UCAI (ulcerative colitis activity index) [21], respectively. The IBD subtype was defined as the Montreal classification [22]. This study was approved by the Ethics Committee of The Fifth Affiliated Hospital, Sun Yat-sen University (L037-1).

### Bacterial cultivation and inflammation-associated protein treatment

Fresh fecal samples from five healthy individuals were brought into an anaerobic workstation (Whitley, 80% N_2_, 10% CO_2_, and 10% H_2_ at 37°C) immediately to cultivate bacteria [23, 24]. *Bacteroides vulgatus* (ATCC 8482), *Bifidobacterium adolescentis* (ATCC 15703), *Enterocloster bolteae* (ATCC BAA-613), and *Ruminococcus gnavus* (ATCC 29149) were grown using the same culture conditions. In addition to single proteins including S100A8, S100A9, IL-1β, and TNF-α, these bacteria were also treated with different combinations of proteins, including S100A8 + S100A9, S100A8 + S100A9 + IL-1β, and S100A8 + S100A9 + TNF-α. The growth, proteomics, and lipidomics of these species were detected to analyze the alterations of gut microbiome in response to treatments (Supplemental methods and materials).

### In-depth metaproteomic analysis of stool samples

Bacteria in fecal samples (~300 mg) were enriched by differential centrifugation. After optimization, a protein:trypsin w:w ratio of 25 000:1 was employed to deplete dominant proteins [16, 17]. The remaining proteins were further digested using a protein:trypsin w:w ratio of 50–100:1. Peptides were divided into three fractions using SDB-RPS and analyzed using a Orbitrap Fusion Lumos mass spectrometer (Thermo Scientific). PEAKS was employed for protein identification using our previously reported comprehensive database (130 975 891 sequences) comprised of human, microbial, and dietary organisms [7]. The two-step strategy was employed to increase the sensitivity of large database searching [7–9]. Proteins were quantified based on peptide-spectrum matches (PSMs)–based label-free quantification (Supplemental methods).

### Functional and microbial taxonomy analysis

Taxonomic and functional analyses of all identified peptides were performed by Unipept (http://unipept.ugent.be) [25] with equated I and L and advanced missing cleavage handling. Gene Ontology (GO) terms of peptides were used as functional annotations. The relative abundance of functional groups was calculated by normalizing the number of corresponding peptides to the total number of peptides. The microbial taxonomic abundance was calculated by summing up the intensity of corresponding peptides. Proteins from microbiota were annotated using iMetaLab [26] and Uniprot.

### Statistical analysis and data visualization

Proteins were normalized by the sum of the samples before further statistical analysis. Statistical significance was assessed by the Kruskal–Wallis test among three groups (present in at least 50% of samples), with missing values replaced by a 0.2-fold minimum value by metaboAnalyst [27]. MaAsLin2 was used to compute the *q* value for age adjustment [28]. Taxonomy–function interactions were generated using iMetaLab. Partial least squares discriminant analysis (PLS-DA) and correlation analysis were performed using RStudio (4.4.2). The interaction networks were calculated by Spearman’s rank correlation (false discovery rate (FDR) < 0.25) and visualized using Cytoscape. Heatmaps, Venn diagrams, and principal coordinate analysis (PCoA) were generated by ImageGP [29].

## Results

### Enhanced metaproteomics profiling of gut ecosystem using a depletion-based approach

**Figure 1:**
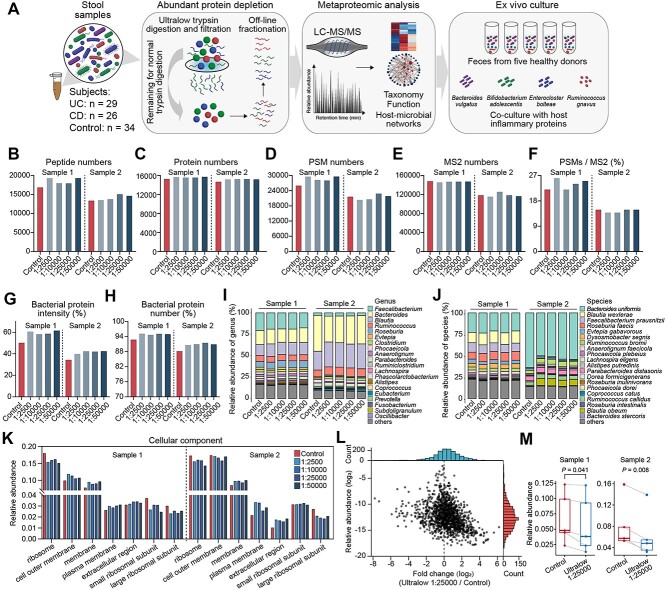
Deep metaproteomic using ultralow trypsin concentration digestion and *ex vivo* microorganism culture for host–microbial interaction analysis. (A) The workflow of depletion-assisted deep metaproteomics and *ex vivo* culture experiments. Microbial proteins were digested with ultralow concentration trypsin and filtered to remove high-abundance peptides. The remaining proteins were subjected to normal trypsin digestion and off-line fractionation for downstream metaproteomic analysis. The *ex vivo* microorganism culture models were incubated with inflammatory proteins to evaluate the host–microbial interaction. (B–M) The performance of the ultralow trypsin concentration digestion method. Total peptide (B) and protein (C) numbers in samples digested with normal method and gradient ultralow trypsin concentration treatments in two samples. Total peptide-spectrum match (PSM) numbers (D), MS/MS (MS2) numbers (E), and PSMs/MS2 ratios (F) in different treatment groups. The percentages of bacterial protein intensity (G) and protein number (H). The relative abundance of bacterial taxonomy identified by peptides at the genus (I) and species level (J). (K) Comparison of bacterial cellular component enrichment based on peptides in ultralow and control groups. (L) Plots of the fold change of proteins (ultralow 1:25 000 to control groups, *n* = 2) versus the relative abundance of proteins in control. Data points are plotted on the basis of protein peak area from two biological replicates. (M) Relative abundance changes of the top five proteins from ultralow 1:25 000 and control group in sample 1 (*P* = .041) and sample 2 (*P* = .008), two-sided paired *t*-test.

We first optimized depletion-based deep stool metaproteomics by comparing different ultralow concentrations (1:2500, 1:10 000, 1:25 000, and 1:50 000 of trypsin/protein ratios) to predigest and deplete high-abundance proteins in two samples (Fig. 1A). The predigestion groups identified more peptides (4.9%–12.2%; Fig. 1B) and proteins (2.8%–3.2%; Fig. 1C) compared to standard condition (no predigestion). Besides, the number of PSMs (8.1%), MS2 (2.4%), and PSMs/MS2 (8.1%) were increased in some cases (Fig. 1D–F). The relative proportion of bacterial proteins also increased in terms of total intensity (18.1%–22.7%) (Fig. 1G) and numbers (2.6%–3.1%) (Fig. 1H). In addition, depletion of high abundance proteins maintains the overall relative abundance of most taxonomic groups (Fig. 1I and J). The bacterial relative abundance of depletion and conventional digestion method was comparable in the mock community experiment (Fig. S1, Table S2), which further revealed the accuracy of predigestion methods. The depleted microbial proteins were mainly from dominant microbial groups such as *Faecalibacterium* and *Bacteroides*. Generally, depletion reduced the relative abundance of high-abundance cellular compartments (Fig. 1K), such as ribosome (by 9.2%–16.8%), small ribosomal subunit (6.5%–17.4%), and large ribosomal subunit (19.3%–26.9%). In contrast, plasma membrane (17.3%–34.8%), cell outer membrane (1.3%–9.2%), membrane (12.8%–20.6%), and extracellular region (32.3%–42.7%) were significantly increased. Cell outer membrane, the interaction zone with environment, as well as extracellular region can exhibit direct interactions with host proteins. Thus, depletion could reveal more host–microbial interaction signatures. Considering the overall performance, a ratio of 1:25 000 was employed for subsequent analysis of IBD samples.

Low-abundance proteins were significantly enriched after depletion (Fig. 1L), whereas the top five most abundant proteins decreased by 24.0% in sample 1 (*P* = .041) and 25.9% in sample 2 (*P* = .008), respectively (Fig. 1M). After depleting high-abundance proteins, we further increased the depth of metaproteomics using off-line fractionation (Figs S2 and S3), increasing the number of identified protein groups from 19 631 to 35 278 (79.7% increase, *n* = 3) (Fig. S3a).

### Disturbed microbial taxonomy and functions in inflammatory bowel disease

**Figure 2:**
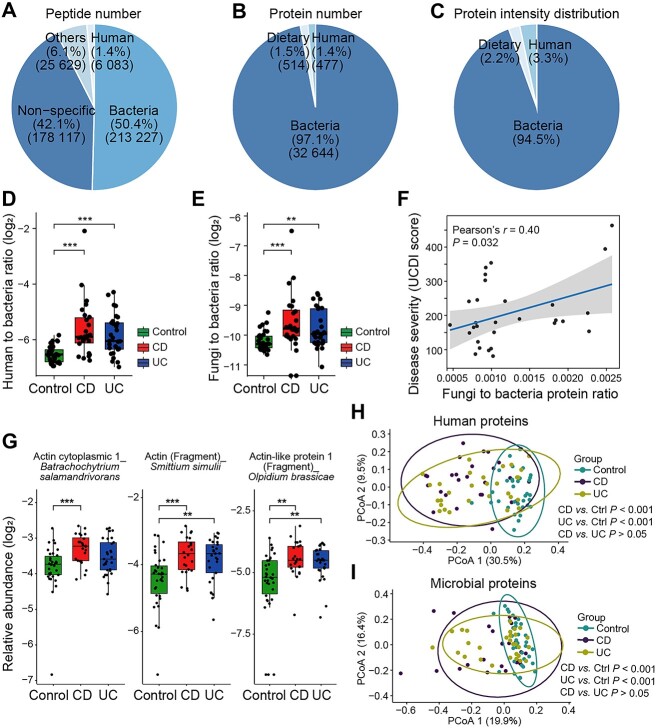
Overview of host and microbial proteome profiling in IBD patients. (A) Total peptide number identified in all samples and the percentage of different organism in metaproteomics. (B) Protein numbers quantified in human, microbiome and dietary organism. (C) Percentage of protein intensity distribution. (D) Boxplot of human to bacteria ratio (log_2_-transformed) based on protein number. ^*^P < .05, ^*^^*^P < .01, ^*^^*^^*^P < .001, Kruskal–Wallis test with post hoc Dunn–Bonferroni analysis. (E) Relative abundance of fungi to bacteria (log_2_-transformed). ^*^P < .05, ^*^^*^P < .01, ^*^^*^^*^P < .001, Kruskal–Wallis test with post hoc Dunn–Bonferroni analysis. (F) Pearson’s rank correlation coefficient *r* of fungi abundance and disease severity scores. CDAI scores for CD and UCAI scores for UC were used to determine disease severity. (G) Discriminant fungal proteins between IBD and control (Kruskal–Wallis test (FDR < 0.05) and MaAsLin2 to adjust age between two groups, ^*^q < 0.05, ^*^^*^q < 0.01, ^*^^*^^*^q < 0.001). Principal coordinates analysis (PCoA) analysis of host proteins (H) as well as microbial proteins (i) based on Bray–Curtis distance. Ellipses represent a 95% confidence interval for each group. Statistics was calculated using pairwise PERMANOVA analyses with the function adonis from the vegan package.

We applied the optimized method to characterize the gut ecosystem of IBD, including 29 UC, 26 CD, and 34 control subjects (Table S3). Consistent with previous reports [30, 31], the majority of CD patients exhibited an ileocolonic location (73.1%, L3) and were in the age group of A2 (73.1%, 17–40 years). Half of the CD patients (50.0%) had the complication of perianal disease modifier. A total of 423 056 peptides and 33 653 protein groups were identified in the metaproteomes of 89 samples with good reproducibility (*r* = 0.86, Pearson’s correlation of QC; Fig. S4). Among all identified peptides, 213 227 (50.4%) and 6083 (1.4%) peptides were annotated to bacteria and human by Unipept, respectively (Fig. 2A). A total of 32 644 (97.1%), 477 (1.4%), and 514 (1.5%) proteins were annotated to be derived from microbial, human, and dietary organisms (Fig. 2B), with their relative intensities accounting for 94.5%, 3.3%, and 2.2%, respectively (Fig. 2C). The human-to-bacteria protein ratio increased in CD (*P* < .001) and UC (*P* < .001) compared to control group (Fig. 2D). Furthermore, the fungi-to-bacteria protein ratio also increased in CD (*P* < .001) and UC (*P* = .001) (Fig. 2E) and was related to UC severity (*r* = 0.40, *P* = .032, Pearson’s correlation; Fig. 2F). Actin of three fungi species including *Batrachochytrium salamandrivorans*, *Smittium simulii*, and *Olpidium brassicae* were increased in IBD (Fig. 2G). Both host and microbial proteome showed significant separation between IBD and control group (*P* < .001) by PCoA (Fig. 2H and I).

**Figure 3:**
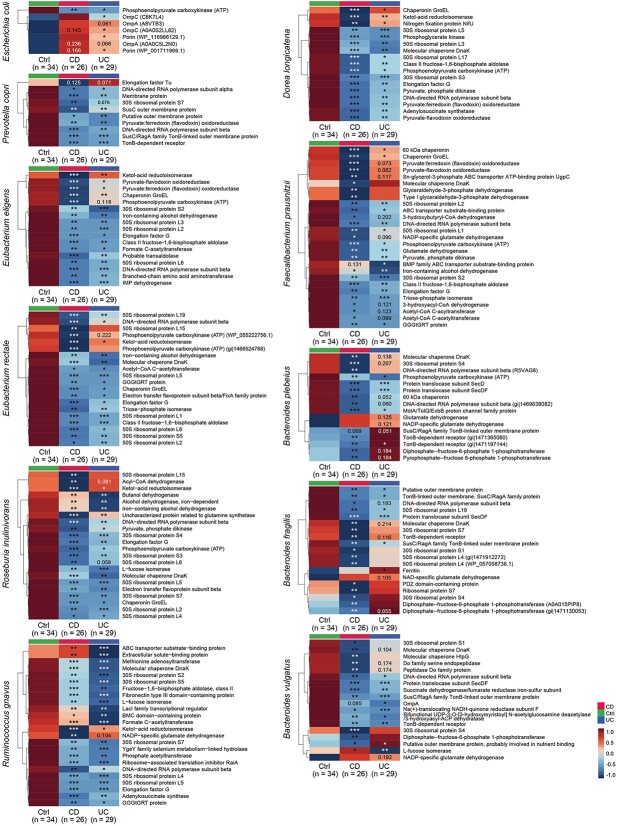
Bacterial protein alterations of eleven main species in IBD and control groups. Significant changes were evaluated by MaAsLin2 (*q* < 0.25) to adjust age between two groups after Kruskal–Wallis test (FDR < 0.05). The colors in the heatmap represent the average of relative abundance in each group. ^*^q < 0.05 versus control; ^*^^*^q < 0.01 versus control; ^*^^*^^*^q < 0.001 versus control.

Protein analysis revealed 8237 discriminant bacterial proteins (Table S4) between IBD and control groups [Kruskal–Wallis test (FDR < 0.05) and MaAsLin2 (*q* < 0.25)]. Eleven species exhibited the most discriminant proteins that were decreased in IBD (Fig. 3), including many house-keep proteins (ribosomal proteins and chaperones) and metabolic enzymes (ketol-acid reductoisomerase and fructose-1,6-bisphosphate aldolase). In contrast, certain membrane proteins of *Escherichia coli* (porin, OmpA, and OmpC) were increased in IBD, facilitating substance transport associated with virulence, host tissue damage, and resistance to antibiotics [32].

**Figure 4:**
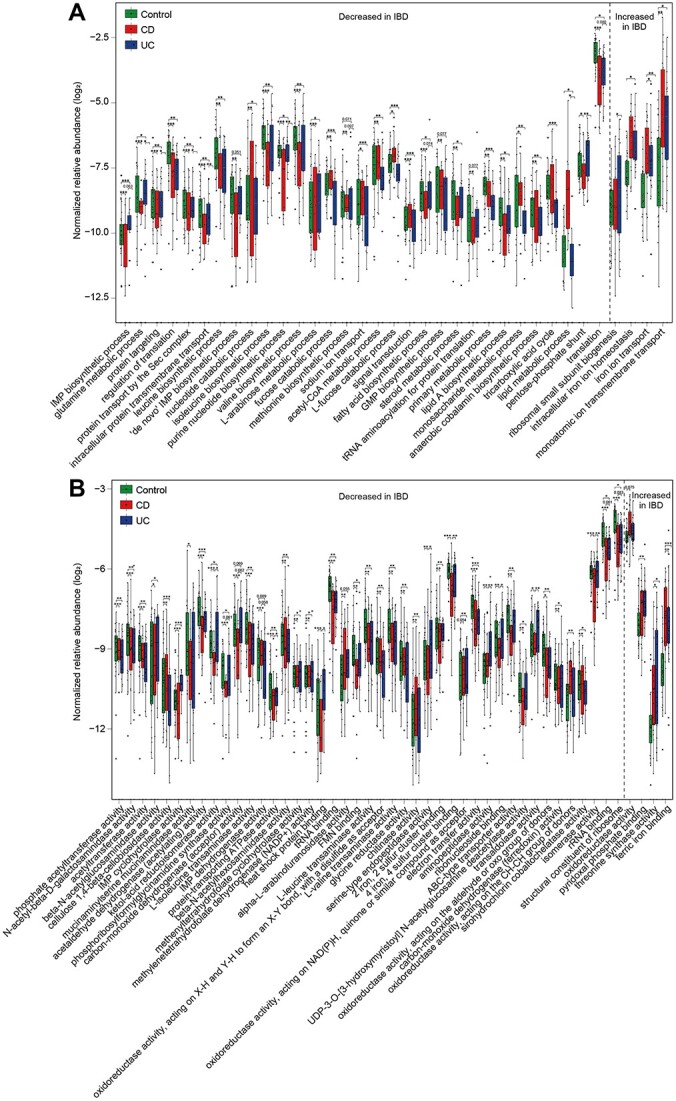
Microbial function alterations in IBD patients. Normalized relative abundance (log_2_-transformed) of altered biological processes (A) and molecular functions (B). Kruskal–Wallis test was employed to evaluate statistical difference among three groups (control, CD, and UC). Function with FDR < 0.05 are shown. The *q* value between two groups was calculated by MaAsLin2 to adjust age. Box plots indicate the first (bottom line), medium (central line), and third (top line) quartiles of the data. Samples are shown as dots (missing values are not included). Outliers >1.5 times the interquartile range (IQR) are indicated as dots. The left of the dotted line represents decreased functions and the right represents increased functions. ^*^q < 0.05, ^*^^*^q < 0.01, ^*^^*^^*^q < 0.001.

In taxonomy analysis, many beneficial short-chain fatty acid (SCFA) producers (for example, family *Lachnospiraceae* and *Clostridiaceae*, genera *Blauria*, *Roseburia*, and *Ruminococcus* [33–35]) decreased, whereas potential pathogenic bacteria, especially members of *Pseudomonadota* and *Actinomycetota*, increased in IBD (Fig. S5a). Consistent with previous studies [36, 37], *Faecalibacterium*, an abundant, anti-inflammatory genus, also decreased in IBD. Function analysis revealed that 35 biological processes (BPs) and 47 molecular functions (MFs) (Fig. 4A) were significantly different between IBD and control groups. Reduced BPs included nutrient metabolism (such as catabolism of acetyl-CoA, L-arabinose metabolic, and monosaccharide), as well as lipid and amino acid metabolism (lipid A, fatty acid, valine, leucine, and isoleucine biosynthetic processes) (Fig. 4A). In addition, protein translation, targeting, and intercellular transmembrane transport decreased, whereas functions in threonine synthase activity [38], oxidoreductase activity, iron ion transport, and homeostasis increased in IBD.

### Altered core host proteins and associated microorganisms in association networks

Among 477 identified human proteins, 21 proteins significantly increased in IBD were involved in immune response and proteolysis, such as the S100 family protein (S100A8, S100A9, S100A12), myeloperoxidase (MPO), immunoglobulin (IGHG1, IGKC), cathepsin G (CTSG), azurocidin (AZU1), and leukocyte protease inhibitor (SERPINB1, SERPINA3) (Fig. S6). Most of these proteins were also positively correlated with disease severity in CD and/or UC (*P* < .05, Spearman’s rank correlations; Fig. S6b). Fecal calprotectin (S100A8/A9) has been reported as a marker of disease activity and histological severity, as well as predicting mucosal healing in IBD patients [39, 40]. Decreased 37 host proteins in IBD were mainly involved in epithelial cell homeostasis and catalytic activity in intestine, such as the mucin (MUC2) [41] and pancreatic alpha-amylase (AMY2A, AMY2B).

**Figure 5:**
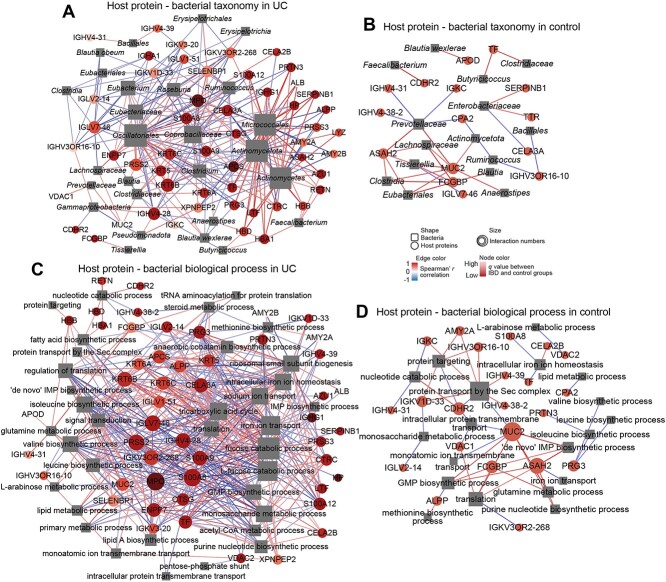
Co-occurrence networks of host proteins and microbiome that were differentially expressed in UC and control. Correlations of differential host proteins and altered gut microbial composition in UC (A) and control (B) (FDR < 0.25). Correlations of differential host proteins and altered microbial biological processes in UC (C) and control (D) (FDR < 0.25). The correlations in networks are calculated by Spearman’s rank correlation (FDR < 0.25). The circle indicates human proteins, and the square indicates microbiome. The size and color of nodes are proportional to the connection number (degree) and *q* value, respectively. The host protein nodes in control are shown in the same color. The edge color is proportional to the Spearman’s rank correlation.

We performed differential host proteins and microbiota correlation analysis to reveal potential host–microbial interactions (Figs 5A and B and S7, Table S5). The central node host proteins in controls were MUC2, *N*-acylsphingosine amidohydrolase 2 (ASAH2), apolipoprotein D (APOD), and carboxypeptidase A2 (CPA2) (Fig. 5B). In contrast, the core host proteins in the association networks of CD and UC groups were proteases CTSG, Chymotrypsin-like elastase family member 3A (CELA3A), and SERPINB1 (Figs 5A and S7c). In addition, UC exhibited several unique core host proteins compared to the control, including myeloperoxidase (MPO), protease serine 2 (PRSS2), and inflammatory proteins S100A8 and S100A9.

Inflammation can reshape gut bacterial composition [42]. S100A9, playing a prominent role in the regulation of inflammatory processes and immune responses, did not exhibit any significant correlation with gut bacteria in controls. However, S100A9 was positively associated with many potential pathogenic bacteria in UC, such as *Actinomycetes*, *Actinomycetota*, and *Micrococcales*, and *Micrococcales* was related to UC severity (Fig. S8a). S100A9 in UC could also suppress beneficial gut bacteria in the *Firmicutes* phylum based on its negative correlations with *Roseburia* (butyric acid producer), *Blautia* (with potential probiotic properties), *Coprobacillaceae*, and *Oscillatoriales*.

In the control group, the probiotic *Blautia wexlerae* was positively associated with apolipoprotein D (APOD). However, such associations were lost in IBD. Compared with other groups, a unique feature of core bacteria in the network of UC was *Coprobacillaceae*, which was positively associated with host chymotrypsin-C (CTRC), CELA3A, PRSS2, and KRT5 and negatively associated with inflammatory proteins, such as MPO and S100 family proteins (S100A8, S100A9, and S100A12), suggesting this gut species may play a role in the pathogenesis of UC (Fig. 5A).

### Disease-specific networks of host–microbial protein functional associations

Host–microbial functional association networks also exhibited a disease-specific pattern in terms of nodes and the links between nodes (positive or negative) (Fig. 5C and D, S7, and Table S5). For instance, homeostasis of branched-chain amino acids (BCAAs) (leucine, isoleucine, and valine) is essential for mammalian health. BCAAs cannot be synthesized endogenously by human but can be produced by gut bacteria. In the control group, microbial biosynthesis of BCAAs was negatively associated with human proteoglycan 3 (PRG3) and myeloblastin (PRTN3) (Fig. 5D). However, microbial BCAAs biosynthesis was positively associated with host proteases Ectonucleotide pyrophosphatase/phosphodiesterase family member 7 (ENPP7), CELA3A, and keratin (KRT6A, KRT6B, and KRT5) in UC. In most cases, host inflammation–associated proteins S100A9 and S100A12 were negatively associated with the majority of gut microbial functions such as monosaccharide metabolic and purine nucleotide biosynthetic process, suggesting inflammation can suppress the metabolism of gut microbiome. Three important exceptions were threonine biosynthesis, intracellular iron ion homeostasis (positively related to CD), and ferric iron binding, which were positively correlated with S100A9 and/or S100A12 (Fig. 5C, S7a and b, and S8b, Table S5). Threonine is essential for intestinal mucin synthesis and threonine-rich glycoproteins production, which can protect mucosal epithelium from injury. In the animal model, more threonine was required when inflammation existed in the intestine [38]. Another exception was that isomerase activity was negatively associated with many host proteins in both CD and UC [for example, S100A8, S10012, MPO, serum amyloid P-component (APCS), and tissue factor (TF)], but this function was only positively associated with MUC2 in the control group. Additionally, intracellular iron ion homeostasis (positively related to CD severity) (Fig. S8b) exhibited a positively correlation with S100A8 and TF in UC, but was negatively associated with host proteins in control.

Mucus layers are the frontline of host–gut bacteria interaction. Host-secreted gel-forming mucin MUC2 was positively correlated with translation, Guanosine-5'-Monophosphate (GMP) biosynthesis, and ribonucleotide binding in control (Figs 5D and S7b), but there was no such association in UC (Figs 5C and S7A). These results suggest distributed host–microbial functional interactions in the mucus layer in IBD.

### *In vitro* gut microbiota treated with S100A8 and cytokines partially resemble inflammatory bowel disease stool metaproteomic alterations

**Figure 6:**
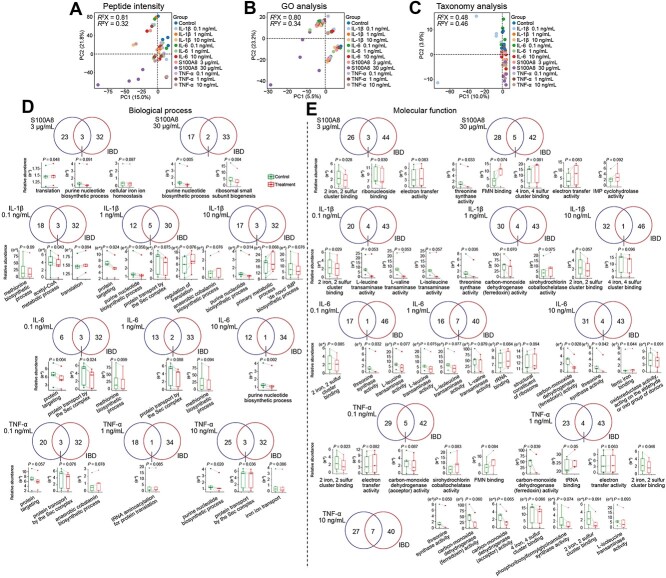
Effect of inflammatory protein on *ex vivo* microorganism culture isolated from five human-fecal samples. (A–C) PLS-DA analysis of microbiome from different treatment groups including S100A8 (3, 30 μg/ml), IL-1β (0.1–10 ng/ml), IL-6 (0.1–10 ng/ml), and TNF-α (0.1–10 ng/ml) based on peptide intensity (A), GO analysis (B), and taxonomy (C). Venn diagrams of discriminant biological processes (D) and molecular functions (E) identified in inflammatory protein treatment (paired *T* test, *P* < .1) and IBD groups (FDR < 0.05). The overlapped functions were further showed as boxplots in inflammatory protein treatment groups.

**Figure 7:**
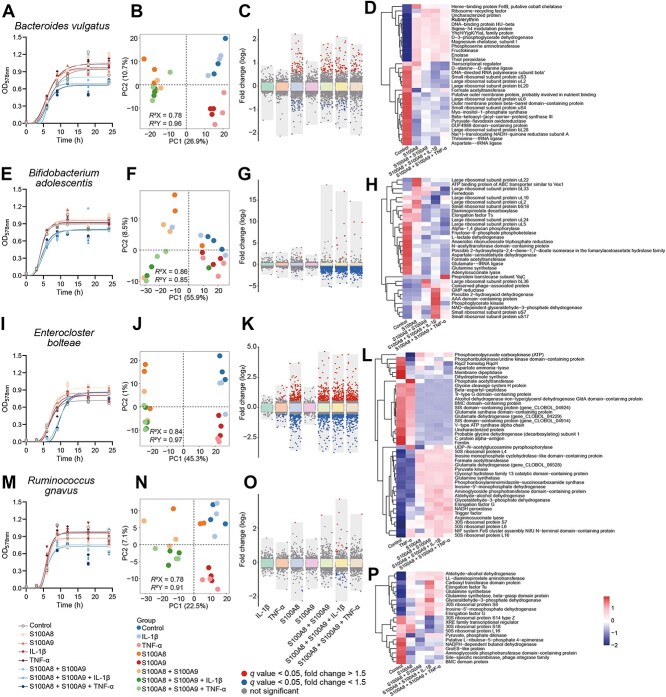
Effect of inflammatory protein on *ex vivo* culture of bacteria including *Bacteroides vulgatus* (A–D), *Bifidobacterium adolescentis* (E–H), *Enterocloster bolteae* (I–L), and *Ruminococcus gnavus* (M–P). The growth curve of *ex vivo* culture of bacteria including *B. vulgatus* (A), *B. adolescentis* (E), *Enterocloster bolteae* (I), and *Ruminococcus gnavus* (M). PLS-DA analysis of microbiome from different treatment groups including IL-1β (10 ng/ml), S100A8 (30 μg/ml), S100A9 (30 μg/ml), TNF-α (10 ng/ml) and different treatment combinations in *B. vulgatus* (B), *B. adolescentis* (F), *E. bolteae* (J), *R. gnavus* (N). The discriminant proteins identified in inflammatory protein treatment group compared to control group (*n* = 3, *q* < 0.05, 1.5-fold change cutoff) in *B. vulgatus* (C), *B. adolescentis* (G), *E. bolteae* (K), *R. gnavus* (O). The top 10 representatively upregulated and downregulated proteins in *B. vulgatus* (D), *B. adolescentis* (H), *E. bolteae* (L), *R. gnavus* (P) (*q* < 0.05).

We further investigated the influence of S100A8, a key host–microbial association protein in UC (Fig. 5A) on stool-derived *in vitro* gut microbial culture derived from five healthy individuals. We also compared them with pro-inflammatory cytokines increased in IBD patients, including IL-1β, IL-6, and TNF-α. Protein concentrations were selected based on previous *in vitro* assays and their physiological concentrations in fecal samples [43–45]. Partial least squares discriminant analysis (PLS-DA) of metaproteomics data revealed that S100A8 (30 μg/ml) treatment induced the most significant alterations based on peptide and GO analysis compared to different concentrations of cytokines (Fig. 6A and B). However, taxonomy analysis indicated that IL-1β was more divergent than other groups in PC1 dimension (Fig. 6C). Some microbial functional alterations of *in vitro* culture induced by these inflammation-associated proteins (paired *T*-test, *P* < .1, Table S6) were also observed in IBD stool samples (FDR < 0.05, Figs 6D and 7E). All tested human proteins (S100A8, IL-1β, IL-6, and TNF-α) at diverse concentrations suppressed microbial purine nucleotide biosynthesis process, which was also reduced in IBD stool samples (Figs 4A and 6D). In addition, protein transport by the Sec complex was inhibited by different cytokines (but not S100A8) and reduced in IBD stool samples. In addition, some reverse trends were also observed in S100A8 and cytokines treated *in vitro* samples compared with IBD stool samples, where threonine synthase was increased in IBD and decreased in vitro (Fig. S6a and e).

### S100A8, S100A9, and cytokines synergistically or antagonistically alter gut bacteria intracellular proteome

To get deeper insights into the effects of host inflammation-associated proteins (S100A8, S100A9, IL-1β, and TNF-α) on gut bacteria, we further investigated the proteome response of four representative species relevant to IBD, including *B. vulgatus*, whose proteases link with UC disease severity [46], *Bifidobacterium adolescentis*, a SCFA producer decreased in IBD [3], *Enterocloster bolteae*, and *Ruminococcus gnavus*, which is associated with CD [47]. We evaluated the effect of host proteins on bacterial growth rate (Table S7), in which S100A8 + S100A9, S100A8 + S100A9 + IL-1β, and S100A8 + S100A9 + TNF-α reduced the total bacterial abundance of *B. vulgatus* (Fig. 7A), *B. adolescentis* (Fig. 7E), and *R. gnavus* (Fig. 7M). In *E. bolteae*, these combination treatment groups inhibited the growth rate rather than the final bacterial abundance (Fig. 7I). However, no such inhabitation effects were observed in the separate IL-1β, TNF-α, S100A8, or S100A9 treatment groups of these bacteria. In proteomic analysis, similar to the above stool *in vitro* culture results, S100A8 induced the most distinct proteomic alterations compared to S100A9, IL-1β, and TNF-α (Fig. 7, Tables S6 and S8). S100A8 (but not the other three proteins) could be fully separated from control in the PC1 dimension in PLS-DA and produced much more differential proteins.

For *B. vulgatus*, the number of altered proteins was generally comparable in S100A8, S100A8 + S100A9, and S100A8 + S100A9 + IL-1β, ranging from 57 to 66, but dropped to 37 in S100A8 + S100A9 + TNF-α (*q* < 0.05, 1.5-fold change cutoff, Fig. 7C). Hierarchical clustering indicated that among the top increased proteins, heme-binding protein FetB exhibited the most divergent expression levels across different groups, which increased 2.6-fold in S100A8, dropped to 1.7-fold increase in S100A8 + S100A9 and S100A8 + S100A9 + IL-1β, but did not alter in S100A8 + S100A9 + TNF-α (Fig. 7D, Table S8). Similarly, many proteins simultaneously increased in S100A8, S100A8 + S100A9, and S100A8 + S100A9 + IL-1β such as lactoyl gluthatione lyase, deoxyuridine 5′-triphosphate nucleotidohydrolase, and 6-phosphogluconate dehydrogenase were not disturbed in S100A8 + S100A9 + TNF-α (Table S8), indicating a potential antagonism between TNF-α and S100A8 + S100A9.

For *B. adolescentis*, S100A8 + S100A9, S100A8 + S100A9 + IL-1β, and S100A8 + S100A9 + TNF-α potently inhibited this species with a global down-regulation of a large number of proteins involved in different biological processes such as translation, transcription, cell division, and regulation of cell shape (Fig. 7G, Table S9). The number of altered proteins increased from 8 in S100A8 to 311 in S100A8 + S100A9, 277 in S100A8 + S100A9 + IL-1β, and further increased to 362 in S100A8 + S100A9 + TNF-α (Fig. 7G). Compared with S100A8 and S100A8 + S100A9, increased GMP reductase, large ribosomal subunit protein bL36, and conserved phage-associated protein were only triggered by S100A8 + S100A9 + IL-1β and S100A8 + S100A9 + TNF-α. For *E. bolteae*, the number of altered proteins increased significantly from 32 in TNF-α to 294 in S100A8, and further increased to 313 in S100A8 + S100A9, 323 in S100A8 + S100A9 + IL-1β and 324 in S100A8 + S100A9 + TNF-α. Several proteins were specifically altered in only one or two groups, such as phosphate acetyltransferase (increased by TNF-α) and UDP-N-acetylglucosamine pyrophosphorylase (increased by TNF-α and S100A8 + S100A9 + IL-1β) (Fig. 7L). For *R. gnavus*, S100A8, S100A8 + S100A9, and S100A8 + S100A9 + TNF-α only resulted in three to four altered proteins, but S100A8 + S100A9 + IL-1β triggered a 9-fold increase in the number of altered proteins (Fig. 7O). These results indicate that S100A8 + S100A9 and IL-1β or TNF-α also play synergistic effects on gut bacteria proteome.

### S100A8, S100A9, and cytokines synergistically regulate *B. vulgatus* lipidome

To further study the molecular effects of inflammatory proteins, we also investigated the lipidome in the bacteria pellets of the above four species. Separate S100A8, S100A9, TNF-α, and IL-1β treatment did not alter gut bacteria lipidome. Disturbed lipids were mainly observed in S100A8 + S100A9 + IL-1β and S100A8 + S100A9 + TNF-α treated *B. vulgatus*, with two groups exhibiting the similar lipid alteration profiles (Fig. S9, Table S10), indicating S100A8 + S100A9 and cytokines synergistically regulate *B. vulgatus* lipids. Gut bacteria also differed in their major altered lipid classes: phosphatidylcholine (PC) and phosphatidylethanolamine (PE) in *B. vulgatus*, monogalactosyldiacylglycerol (MGDG) in *B. adolescentis*, diacylglycerol (DG) and phosphatidylinositol (PI) in *E. bolteae*, and diacylglycerol (DG) and digalactosyldiacylglycerol (DGDG) in *R. gnavus*.

### S100A8, S100A9, and cytokines synergistically or antagonistically alter gut bacteria secretome

**Figure 8:**
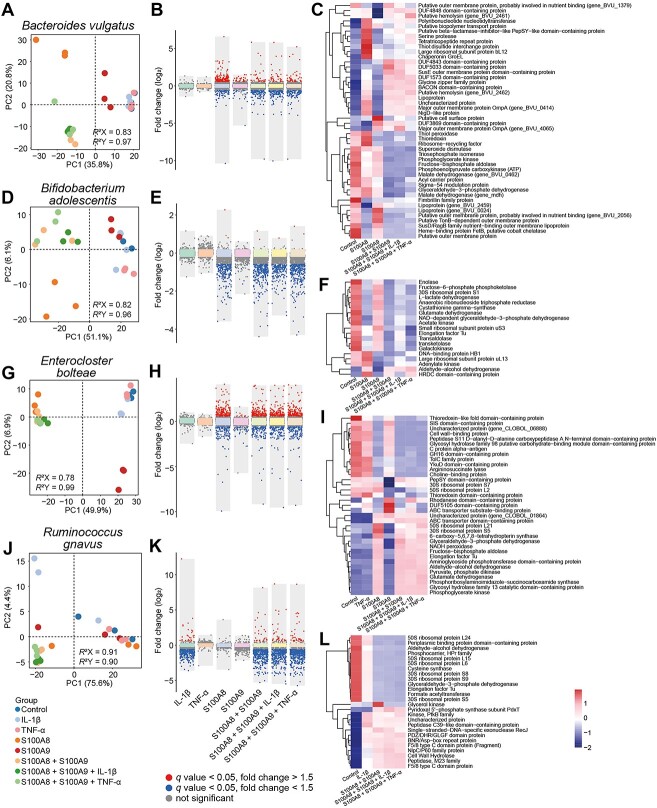
Effects of inflammatory protein on bacterial secretory proteins in *Bacteroides vulgatus* (A–C), *Bifidobacterium adolescentis* (D–F), *Enterocloster bolteae* (G–I), and *Ruminococcus gnavus* (J–L). PLS-DA analysis of microbiome from different treatment groups including IL-1β (10 ng/ml), S100A8 (30 μg/ml), S100A9 (30 μg/ml), TNF-α (10 ng/ml) and different treatment combinations in *B. vulgatus* (A), *B. adolescentis* (D), *E. bolteae* (G), *R. gnavus* (J). The discriminant proteins identified in inflammatory protein treatment group compared to control group (*n* = 3, *q* < 0.05, 1.5-fold change cutoff) in *B. vulgatus* (B), *B. adolescentis* (E), *E. bolteae* (H), and *R. gnavus* (K). The top 10 representatively upregulated and downregulated proteins in *B. vulgatus* (C), *B. adolescentis* (F), *E. bolteae* (I), *R. gnavus* (L) (*q* < 0.05).

We analyzed the bacterial secretory protein alterations (Fig. 8, Tables S11 and S12). PLS-DA scatter plots (Fig. 8A, D, F, and J) revealed a distinct separation between S100A8 and control in both PC1 and PC2 dimensions in *B. vulgatus*, *B. adolescentis*, and *E. bolteae* with a large number of altered proteins (Fig. 8B, E, H, K). S100A9 triggered secretory protein alterations of *B. vulgatus* and *E. bolteae*, whereas IL-1β only alterated *R. gnavus* secretome. In all cases, TNF-α did not significantly altered the secretome of the studied four gut bacteria and clustered with the control group in PLS-DA. For *B. vulgatus*, combing S100A9, TNF-α, or IL-1β with S100A8 reduced the number of significantly increased proteins from 137 in S100A8 to 29–38 in the combination groups, whereas increased the number of significantly reduced proteins from 68 to 94–101 (Fig. 8B). This phenomenon was not observed in *B. adolescentis*, *E. bolteae*, or *R. gnavus*, where the proportions of increased or decreased proteins were comparable in different groups (Fig. 8E, h, and K).

For *B. vulgatus*, heme-binding protein FetB (involved in heme capture) was the most downregulated secretory proteins in all cases, a 39-fold reduction in S100A9 and an 888–1325-fold reduction in other groups (Fig. 8C, Table S11). S100A8 + S100A9 and their combinations with TNF-α or IL-1β (but not separate S100A8 or S100A9) significantly reduced magnesium chelatase, subunit I (Table S11**)**. Similarly, S100A9, S100A8 + S100A9, S100A8 + S100A9 + TNF-α, and S100A8 + S100A9 + IL-1β but not separated S100A8 significantly reduced antioxidant proteins such as thiol peroxidase and superoxide dismutase. Additionally, S100A9 significantly reduced the exotoxin hemolysin, S100A8, S100A8 + S100A9, S100A8 + S100A9 + TNF-α, and S100A8 + S100A9 + IL-1β all increased this toxic factor.

Similar to the intracellular proteome (Fig. 7E), a large number of secretory proteins of *B. adolescentis* were also down-regulated by S100A8 + S100A9, S100A8 + S100A9 + IL-1β, and S100A8 + S100A9 + TNF-α (Fig. 8E and F). Although *R. gnavus* intracellular proteins did not significantly altered by the studied inflammatory proteins (Fig. 7K), a large number of its secretory proteins were disturbed by IL-1β (*n* = 275), S100A8 + S100A9 (*n* = 347), S100A8 + S100A9 + IL-1β (*n* = 350), and S100A8 + S100A9 + TNF-α (*n* = 347) (Fig. 8K, Table S11). In all cases, the largest increase was peptidase C39-like domain-containing protein (Fig. 8L). Other major increased proteins were F5/8 type C domain protein (carbohydrate-binding) and different hydrolases, including NlpC/P60 family protein (peptidoglycan hydrolase), peptidase M23 family, PDZ/DHR/GLGF domain protein (possibly a glycosyl hydrolase), and cell wall hydrolase. The studied inflammatory proteins also triggered an opposite alteration in pyridoxal 5′-phosphate synthase subunit PdxT (involved in vitamin B6 metabolism), which increased 11.7-, 30.9-, and 16.6-fold by S100A8 + S100A9, S100A8 + S100A9 + IL-1β, and S100A8 + S100A9 + TNF-α, respectively, but decreased by 50% in the IL-1β group. The major decreased proteins were essentially consistent in the above four groups, such as ribosomal proteins, formate acetyltransferase, and phosphocarrier, HPr family.

Compared with the other three species, a unique alteration of *E. bolteae* secreted proteins was choline-binding protein, which exhibited a 6.6-fold reduction in S100A9, and a 38.1–40.0-fold reduction in S100A8, S100A8 + S100A9, S100A8 + S100A9 + IL-1β, and S100A8 + S100A9 + TNF-α. Choline, present in a variety of foods, is an essential nutrient for the host. Host–microbial cometabolism of choline plays an important role in host fitness, and certain metabolites such as trimethylamine *N*-oxide (TMAO) may raise the risk of cardiovascular diseases [48]. Our study indicated that *E. bolteae* may be involved in the metabolism of choline and host inflammatory proteins can affect the microbial metabolism of choline by downregulating choline-binding protein.

### Gut bacteria degrade significantly more S100A8 than S100A9 in the presence of both proteins

As a defense mechanism, bacteria can also inhibit or degrade host immune factors. Strikingly, compared to medium containing S100A8 alone, the remaining S100A8 in the media containing both S100A8 and S100A9 (S100A8 + S100A9, S100A8 + S100A9 + IL-1β, and S100A8 + S100A9 + TNF-α) decreased by 79.2–118.1-, 18.0–110.3-, and 146.5–241.4-fold in *B. vulgatus*, *B. adolescentis*, and *E. bolteae*, respectively (Table S13). In contrast, compared to medium containing S100A9 alone, the remaining S100A9 in the media containing both S100A8 and S100A9 increased by 2.5–4.2-, 13.2–42.6-, and 30.4–103.1-fold in *B. vulgatus*, *B. adolescentis*, and *R. gnavus*, respectively, and decreased by 22.1–27.5-fold (a 5.3–9.4-fold lesser degree compared with S100A8 reduction) in *E. bolteae* (Table S13). Taken together, these results suggested that gut bacteria degraded significantly more S100A8 than S100A9 in the presence of both proteins, even though S100A8 was much more potent in stimulating or inhibiting gut bacteria.

## Discussion

We demonstrated that ultralow trypsin predigestion is a straightforward approach to improve the coverage of low-abundance microbial proteins (especially membrane, cell wall, and extracellular proteins) and maintain the overall relative abundance of most taxonomic groups in metaproteomics. This is probably because ultralow trypsin prefers to digest highly abundant host protein–dominated fecal samples, depletion of which results in more chances to sequence less abundant microbial proteins by mass spectrometry.

Network-centric analysis revealed disease-specific host–microbial protein associations in IBD. The core host proteins in the network shifted from those maintaining mucosal homeostasis (MUC2 and ASAG2) in control to those involved in inflammation (S100A8, S100A9, and MPO), proteolysis, and intestinal barrier (KRT6A and KRT6B) in IBD. Many functionally important microbial nodes in the network were largely lost or suppressed in IBD. Our network analysis revealed that inflammatory proteins can promote potential pathogenic bacteria and suppress beneficial gut bacteria in UC. Restoring those key microbial functions and suppressing disease-specific bacteria may contribute to treating IBD. However, our peptide-centric taxonomic and functional analysis may suffer from information loss and misassignments because peptides are generally short and thus are hard to assign to specific proteins. Since the microbial sequences in the database used are not specific to the samples analyzed, the accuracy of the peptide and proteins identified may be impacted. The two-step searching approach could also underestimate FDR. Identification confidence could be improved by combining results from multiple software.

In accordance with our finding that S100A8 and S100A9 were among the core microbiome associated host proteins, fecal calprotectin (S100A8/A9) is both sensitive and specific to IBD disease activity and histological severity surveillance [49]. In addition, pro-inflammatory cytokines (IL-1β, IL-6, and TNF-α) have been reported to be able to bind bacteria and affect the growth rate of *Bifidobacteria longum* GT15 strain and certain gene expression [50–52]. However, the detailed molecular effects of these host inflammatory proteins on gut microorganisms have not been systemically studied by multi-omics approaches. The present metaproteomics study demonstrated that purine nucleotide biosynthesis was suppressed in both IBD stools and *in vitro* stool microbial cultures (derived from healthy subjects) treated with S100A8, IL-1β, IL-6, and TNF-α. Host S100A8/A9 has been reported to induce bacterial metal starvation through chelation of nutrients (zinc and manganese) [53], and can result in robust transcriptional alterations in *E. coli* [54]. Accordingly, our present study revealed that the most downregulated secretory proteins of *B. vulgatus* by these host proteins was heme-binding protein FetB involved in heme capture.

Our study of single gut bacteria revealed that S100A8 + S100A9, S100A8 + S100A9 + IL-1β, and S100A8 + S100A9 + TNF-α suppressed a large number of biological processes of beneficial *B. adolescentis* intracellular and extracellular proteome, indicating these human inflammatory proteins may be involved in the inhibitions of probiotics. IL-1β, S100A8 + S100A9, S100A8 + S100A9 + IL-1β, and S100A8 + S100A9 + TNF-α altered a large number of *R. gnavus* secretory proteins, but not intracellular proteins.

S100A8/A9 is present in ~50% of the neutrophil cytoplasm content and also produced by monocytes/macrophages and potentially epithelial cells [55], and cytokines (IL-1β, IL-6, and TNF-α) can be released by monocytes/macrophages after bacteria and endotoxin stimuli. Furthermore, S100A8 can bind and capture cytokines in a noncovalent manner and inhibit the generation of IL-6 and TNF-α by monocytes [56, 57]. Different host cells can exert synergistic and antagonistic regulation effects on gut bacteria through these secreted proteins. Overall, our study reveals complex interactions between host inflammatory proteins and gut bacteria.

## Supplementary Material

Additional_file_2_wrae146

Additional_file_3_wrae146

Additional_file_4_wrae146

Additional_file_5_wrae146

Additional_file_6_wrae146

Additional_file_7_wrae146

Additional_file_8_wrae146

Additional_file_9_wrae146

Additional_file_10_wrae146

Additional_file_11_wrae146

Additional_file_12_wrae146

Additional_file_13_wrae146

Additional_file_14_wrae146

Supporting_Information_wrae146

## Data Availability

The metaproteomics mass spectrometry proteomics data have been deposited to the ProteomeXchange Consortium (http://proteomecentral.proteomexchange.org) via the iProX partner repository with the dataset identifier PXD043380. The data supporting the conclusions of this study is included in the paper and supplementary materials.
