# Supplementary material for: Core microbiome-associated proteins associated with ulcerative colitis interact with cytokines for synergistic or antagonistic effects on gut bacteria
Source: ISME J. 2024 Jul 29;18(1):wrae146. doi: 10.1093/ismejo/wrae146 (PMC11360980; doi:10.1093/ismejo/wrae146)
Supplement: Supporting_Information_wrae146 [file supporting_information_wrae146.docx]

Supporting Information

**Core microbiome-associated proteins associated with ulcerative colitis interact with cytokines for synergistic or antagonistic effects on gut bacteria**

Running title: Inflammatory proteins alter gut bacteria

Ting Zhang ^a,b#^, Hang Zhong ^a,b#^, Lu Lin ^c#^, Zhiyan Zhang ^a,b#^, Kewen Xue ^a,b^, Feixiang He ^a,b^, Yingshu Luo ^c^, Panpan Wang ^d^, Zhi Zhao ^d^, Li Cong ^d^, Pengfei Pang ^a,b^*, Xiaofeng Li ^c^*, Hong Shan ^a,b^*, Zhixiang Yan ^a,b^*

*^a^ Guangdong Provincial Engineering Research Center of Molecular Imaging, The Fifth Affiliated Hospital, Sun Yat-sen University, and Southern Marine Science and Engineering Guangdong Laboratory (Zhuhai), Meihua East Road, Zhuhai, Guangdong Province, 519000, China*

*^b^ Guangdong-Hong Kong-Macao University Joint Laboratory of Interventional Medicine, The Fifth Affiliated Hospital, Sun Yat-sen University, Meihua East Road, Zhuhai, Guangdong Province, 519000, China*

*^c^ Department of Gastroenterology, The Fifth Affiliated Hospital, Sun Yat-sen University, Meihua East Road, Zhuhai, Guangdong Province, 519000, China*

*^d^ Department of Endocrinology and Metabolism, The Fifth Affiliated Hospital, Sun Yat-sen University, Meihua East Road, Zhuhai, Guangdong Province, 519000, China*

^#^ These authors contributed equally to this work.

* Correspondence: Pengfei Pang, pangpf@mail.sysu.edu.cn; Xiaofeng Li, zdwylxf@163.com; Hong Shan, shanhong@mail.sysu.edu.cn; Zhixiang Yan, yanzhx3@mail.sysu.edu.cn.

**Table of Contents**

1. Supplemental methods and materials.

2. Supplemental Figure S1. Quantification of mock community using conventional digestion and ultralow trypsin digestion methods with five processing replicates.

3. Supplemental Figure S2. The proteome of the three fractions identified by the ultralow method with fractionations.

4. Supplemental Figure S3. The performance of off-line fractionation metaproteomics.

5. Supplemental Figure S4. Sample-sample correlation heatmap calculated with identified metaproteomes across all samples.

6. Supplemental Figure S5. Taxonomical alterations in IBD patients.

7. Supplemental Figure S6. Alterations of host proteome in IBD patients.

8. Supplemental Figure S7. Co-occurrence networks of host proteins and microbiome that were differentially expressed in UC, CD and control.

9. Supplemental Figure S8. Correlations of disease severity and altered microorganisms, biological processes, and molecular functions in UC and CD.

10. Supplemental Figure S9. Bacterial lipidome alterations after inflammatory protein treatment in vitro.

**Supplemental methods and materials**

**Microbiota enrichment by differential centrifugation**

Fecal samples (~300 mg) were suspended in 25 mL of precooled 0.9% NaCl and vortexed thoroughly before being centrifuged at 250 g for 5 minutes at 4℃. The central supernatant (~15 mL) was aspirated into a new tube to remove large dietary debris and the upper lipid layer. Resuspend the remaining pellet in 15 mL of 0.9% NaCl and centrifugate at 250 g at 4℃ for 5 min. The central supernatant was collected and pooled together for another centrifugation at 35 000 g and 4℃ for 30 min. The final pellet was collected for protein extraction.

**Assembly of mock community**

The mock community covering a large range of species abundances was assembled to evaluate the accuracy of the quantification method. *Bacteroides uniformis* ATCC 8492, bovine albumin (BSA, serving as host and food proteins), *Prevotella copri* DSM 18205, *Bacteroides ovatus* ATCC 8483, *Bacteroides vulgatus* ATCC 8482, *Enterococcus faecium* ATCC 19434, *Bacteroides dorei* DSM 17855, *Bifidobacterium adolescentis* ATCC 15703, *Akkermansia muciniphila* ATCC BAA835, *Bacteroides cellulosilyticus* DSM 14838, *Bifidobacterium dentium* ATCC 27534, *Clostridium perfringens* ATCC 13124, *Ruminococcus gnavus* ATCC 29149, *Alistipes indistinctus* DSM 22520, *Bacteroides fragilis* ATCC 25285, *Clostridium bolteae* ATCC BAA-613, *Prevotella stercorea* DSM 18206, *Streptococcus parasanguinis* ATCC 15912, *Lactobacillus jensenii* ATCC 33323, and *Flavonifractor plautii* ATCC 49531 were combined based on the protein biomass ratio of 100:80:40:10:8:5:5:5:2:2:2:2:2:1:1:1:1:1:0.5:0.5. All bacteria were purchased from the Guangdong Microbial Culture Collection Center.

**Protein extraction and purification**

The microbial pellet was suspended with 100 μL lysis buffer containing 10 mM tris(2-carboxyethyl) phosphine (TCEP, Sigma, USA), 40 mM chloroacetamide (CAA, Sigma) and 6M Guanidinium hydrochloride (GdmCI, Sigma) in 100 mM Tris buffer (pH=8.5, Sigma) and homogenized with Zirconia magnetic beads by 3D Cryogenic Grinder (Jingxin, Shanghai, China) in 20 Hz for 10 min at -20℃. The protein supernatant was collected after centrifugated at 16 000 g for 10 min at 4℃ to remove cell debris and precipitated by adding a 0.25-fold volume of precooled trichloroacetic acid (TCA, Sigma) at 4℃ for 50 min. Protein pellets were obtained by centrifuging at 16 000 g for 15 min at 4℃ and washing three times with ice-cold acetone. After acetone dried, protein pellets were resuspended in lysis buffer (6 M GdmCl, 10 mM TCEP, 40 mM CAA, 100 mM Tris, pH 8.5). The protein suspension was then boiled for 15 min at 95℃ and sonicated for 20 min. The protein concentration was measured by A280 in Nanodrop (Thermo Scientific, USA). A quality control (QC) sample containing five randomly selected samples was divided into three samples and subjected to further peptide preparation to make sure consistency in sample preparation and MS performance.

**Digestion and depletion of abundant proteins**

Trypsin (Promega, USA) was added in a gradient (2 500, 10 000, 25 000, and 50 000:1) protein:trypsin mass ratio with a dilution buffer (10% (v/v) acetonitrile (ACN, Thermo Scientific), 25 mM Tris, pH 8.5) to digest for 12 hours at 37℃. These trypsin concentrations were selected based on previous reports [1, 2]. Digests were filtered by multi-well plates (cut off at 3 kDa, AcroPrep Advance 96 Well 350 μL, PALL, USA) equipped with a QIAvac 96 vacuum manifold (QIAGEN GmbH, Hilden, German) until 100-200 μL remained in the plates. The remaining proteins were subjected to normal tryptic digestion (50-100:1 protein:trypsin (w:w)) at 37℃ overnight, which was identical to the control method in this step.

**Peptide desalting and off-line fractionation**

Peptides were acidified by trifluoroacetic acid (TFA, Sigma) to a 1% final concentration and centrifugated at 16 000 g for 15 min to remove debris. Desalting was accomplished using StageTips of assembled C18 or SDB-RPS (poly (styrene divinylbenzene) reverse phase sulfonate, Sigma) materials, according to a previous report [3]. Peptides obtained from C18 have one aliquot. Three fractions from SDB-RPS material were eluted by a stepwise increasing gradient of ACN (40%, 60%, 80%). Purified peptides were dried in a concentrator (Eppendorf, German) at room temperature. Dried peptides from each fraction were resuspended in 20 μL of 2% ACN in 0.1% formic acid (FA) and sonicated for 10 min for LC-MS/MS. The depletion-assisted approach was applied for fecal samples but not in vitro culture samples; because the fecal samples contain a large range of bacteria taxa and host and food proteins, which were significantly more complex than in vitro single-strain bacterial samples.

**In-depth metaproteomic analysis of stool samples**

Peptides from stool samples were loaded onto an Acclaim PepMap 100 C18 column (75 μm × 20 mm, 2 μm, 100 Å, Thermo Scientific) and separated on an Acclaim PepMap 100 C18 column (75 μm × 250 mm, 2 μm, 100 Å, Thermo Scientific) using a a Dionex UltiMate 3000 RSLCnano LC system (Thermo Scientific) at 300 nL/min with gradient solvent A (0.1% formic acid in water) and solvent B (0.1% formic acid in 80% ACN). The separation gradient comprised an increase from 8% to 15% B in 30 min, 15% to 22% in 70 min, 22% to 98% in 95 min, 98% for 10 min and equilibrated at 8% B for the last 10 min. Elutes were introduced into an Orbitrap Fusion Lumos Tribrid mass spectrometer (Thermo Scientific) in positive ion mode with a spray voltage of +2700 V. A full MS scan was acquired from 400 to 1 600 m/z with a resolution of 60 000. MS/MS spectra were acquired in data-dependent acquisition (DDA) using collisional dissociation (HCD) with a resolution of 15 000.

Protein identification was performed using the search engine PEAKS. The protein database, containing a total of 130 975 891 non-redundant sequences, was comprised of human, microbial, and dietary organisms [4]: (1) the integrated human fecal metagenomes' gene catalogue [5], (2) the genomes of more than 6 000 isolated human fecal bacteria [6], (3) the genomes of 215 human fecal bacteria isolates [7], (4) Archaea, Bacteria, and Fungi in UniProtKB (Release 2017_06) and NCBI RefSeq (Release 90), (5) the human proteome (UniProt 2017_06), (6) a food database of common dietary organisms. The mass tolerance of precursor and fragment ions was set to 15 ppm and 0.03 Da, respectively. Trypsin was set to be the cleavage enzyme, allowing a maximum of three missing cleavage sites. Carbamidomethylation of Cys, acetylation of protein N-terminus, Met oxidation, Asn and Gln deamidation, and Pyro-glu from Gln were specified as variable modifications, with a maximum of three per peptide. The two-step strategy was employed to increase the sensitivity of large database searching [4]. Briefly, no FDR threshold was applied in the first step search, where all identified proteins were used for the second step search with a false discovery rate (FDR) set to 1% at both protein and peptide levels.

**Bacterial cultivation and inflammation-associated protein treatment in vitro**

The stool sample (~1 g) was suspended in 20 mL of sterile phosphate buffer (PBS) with 0.1% L-cysteine and 10% glycerol (v/v). The suspension was left standing still for 5 min and filtered using sterile gauze to collect the fecal inoculum. The glycerol stock was stored at -80°C and stabilized at a concentration of 1% (v/v) in Gifu Anaerobic Medium (GAM) for 12 h before coculturing experiments. The GAM was sterilized and uncapped overnight in an anaerobic workstation to remove oxygen in advance. 5 μL of fecal inoculum was cocultured with different combinations of proteins, including IL-1β (PeproTech, USA), IL-6 (PeproTech), TNF-α (PeproTech), S100A8 (MCE, USA), and S100A9 (MCE) in 1 mL of culture medium into a 96-well deep-well plate. The plate was shaken at 250 rpm with a digital shaker (SC 240C, DAM, Shanghai) at 37 °C for 12 h in the anaerobic chamber.

*Bacteroides vulgatus* (ATCC 8482), *Bifidobacterium adolescentis* (ATCC 15703), *Enterocloster bolteae* (ATCC BAA-613), and *Ruminococcus gnavus* (ATCC 29149) were grown anaerobically at 37 °C using the above culture conditions. Growth was monitored by measuring OD578 nm using an Eon Microplate Spectrophotometer (BioTek) every 3 h for the first 12 h, and then approximately every 6 h. In addition to single proteins including S100A8, S100A9, IL-1β, and TNF-α, these bacteria were also treated with different combinations of proteins, including S100A8 + S100A9, S100A8 + S100A9 + IL-1β, and S100A8 + S100A9 + TNF-α. After incubation, cultures were centrifuged (4 000 rpm, 20 min) and the supernatant was retained to analyze bacterial secretory proteins. The remaining pellets were resuspended in 1 mL of precooled PBS and transferred to 1.5 mL tubes to centrifugate at 10 000 g at 4℃ for 10 min. Repeat the washing step twice and collect the microbial pellet.

**Proteomic analysis of** **in vitro culture**

Proteomics of bacterial cell pellets from stool and single bacteria in vitro culture were performed using an Orbitrap Fusion Lumos Tribrid mass spectrometer. Peptides were loaded using Evotip as disposable trap columns and separated on a C18 column (100 μm × 8 cm, 3 μm) using a Evosep One liquid chromatography platform using built-in SPD60 method. Elutes were introduced into the mass spectrometer with a FAIMS Pro duo differential ion mobility interface with compensation voltage (CV) was set to −45. The spray voltage was +2700 V. For in vitro culture of stool samples, data was acquired in DDA mode and analyzed as above. For single bacteria species, data was acquired in data-independent acquisition (DIA) mode and analyzed using Spectronaut (version 15.7.220308.50606) with default settings. Proteomics of single bacteria culture supernatant were performed on a ZenoTOF 7600 in data-independent acquisition (DIA) ZenoSWATH mode and data was analyzed using DIA-NN (version 1.8.1).

**Lipidomics of in vitro culture**

Twenty microliters of bacteria lysate were mixed with 100 μL chloroform and methanol (3:1 v/v). Samples were vortexed for 45 s and centrifuged at 16,000 g for 15 min at 4 °C. The supernatants were collected, and vacuum-dried by Speed Vac, suspended in 30 μ1 chloroform and methanol (5:13 v/v) and centrifuged at 16,000 g for 15 min at 4 °C. 28 μ1 of the supernatant was transferred into the sample vials maintained at 8°C for LC-MS analysis. Two microliters of samples were separated on an ACQUITY UPLC HSS T3 analytical column (2.1 × 50 mm, 1.7 μm, 100 Å, Waters) at 42°C using a 10-min gradient at 0.5 mL/min. Mobile phase A and B were ACN: H_2_O (60:40 v/v) and isopropanol: ACN (90:10 v/v), respectively, both contained 10 mM ammonium acetate and 0.1% acetic acid. LC eluents were introduced into an Orbitrap Exploris 480 mass spectrometer (Thermo Scientific) in both positive and negative ionization modes with a spray voltage of +3500 V and -3000 V, respectively. Data were collected in DDA mode using a full MS scan range of *m/z* 150-2000 at 30K resolution followed by 5 MS/MS scans at 15K resolution with a normalized collision energy (NCE) at 30. Data were processed using LipidSearch 5.0 (Thermo Fisher) with a RT Tolerance of 0.1 min.

**Supplementary references**

1. Fonslow BR, Stein BD, Webb KJ, Xu T, Choi J, Park SK, et al. Digestion and depletion of abundant proteins improves proteomic coverage. Nat Methods. 2013;10(1):54-6.

2. Nie S, Greer T, O'Brien Johnson R, Zheng X, Torri A, Li N. Simple and Sensitive Method for Deep Profiling of Host Cell Proteins in Therapeutic Antibodies by Combining Ultra-Low Trypsin Concentration Digestion, Long Chromatographic Gradients, and BoxCar Mass Spectrometry Acquisition. Anal Chem. 2021;93(10):4383-90.

3. Kulak NA, Pichler G, Paron I, Nagaraj N, Mann M. Minimal, encapsulated proteomic-sample processing applied to copy-number estimation in eukaryotic cells. Nat Methods. 2014;11(3):319-24.

4. Yan Z, He F, Xiao F, He H, Li D, Cong L, et al. A semi-tryptic peptide centric metaproteomic mining approach and its potential utility in capturing signatures of gut microbial proteolysis. Microbiome. 2021;9(1):12.

5. Li J, Jia H, Cai X, Zhong H, Feng Q, Sunagawa S, et al. An integrated catalog of reference genes in the human gut microbiome. Nat Biotechnol. 2014;32(8):834-41.

6. Zou Y, Xue W, Luo G, Deng Z, Qin P, Guo R, et al. 1,520 reference genomes from cultivated human gut bacteria enable functional microbiome analyses. Nat Biotechnol. 2019;37(2):179-85.

7. Browne HP, Forster SC, Anonye BO, Kumar N, Neville BA, Stares MD, et al. Culturing of 'unculturable' human microbiota reveals novel taxa and extensive sporulation. Nature. 2016;533(7604):543-6.


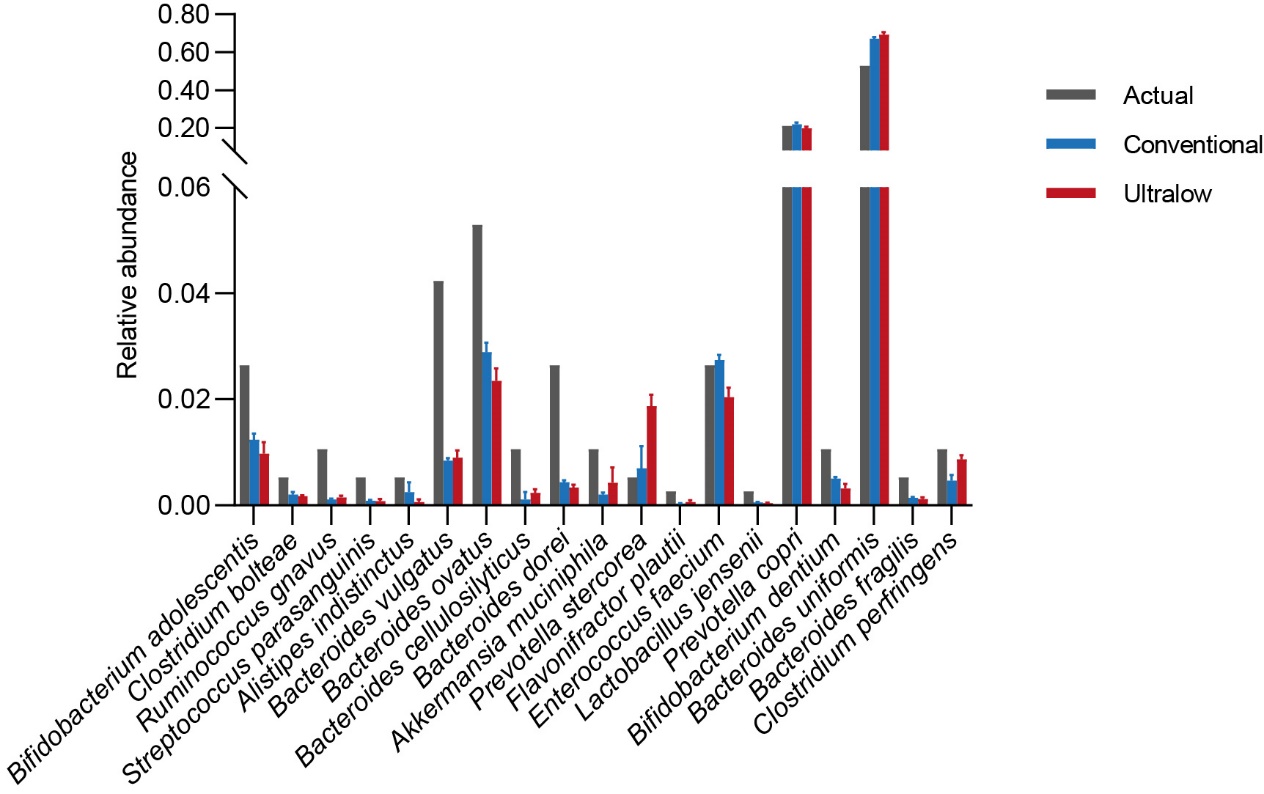


**Figure S1.** Quantification of mock community using conventional digestion and ultralow trypsin digestion methods with five processing replicates. Quantification of different species was based on the sum intensities of their unique proteins.


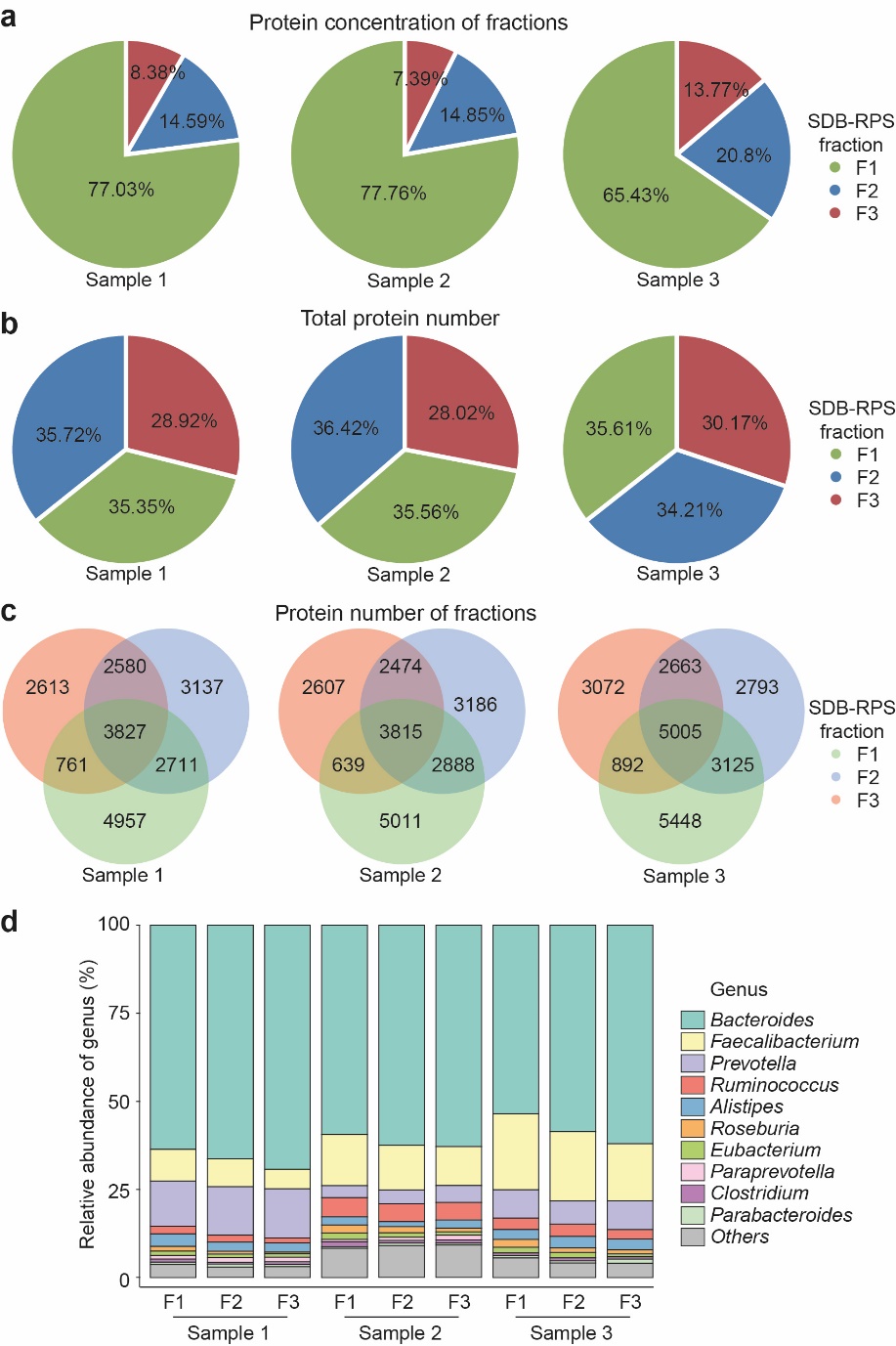


**Figure S2**. The proteome of the three fractions identified by the ultralow method with fractionations. (a) Protein concentration percentages of three fractions obtained by different gradients of organic solvent elution (each circle replicates a replicate). (b) Percentages of protein number identified in three factions. (c) Venn diagrams for total protein numbers among three fractions. (d) The relative abundance of bacteria at the genus level identified in three fractions (three biological replicates).


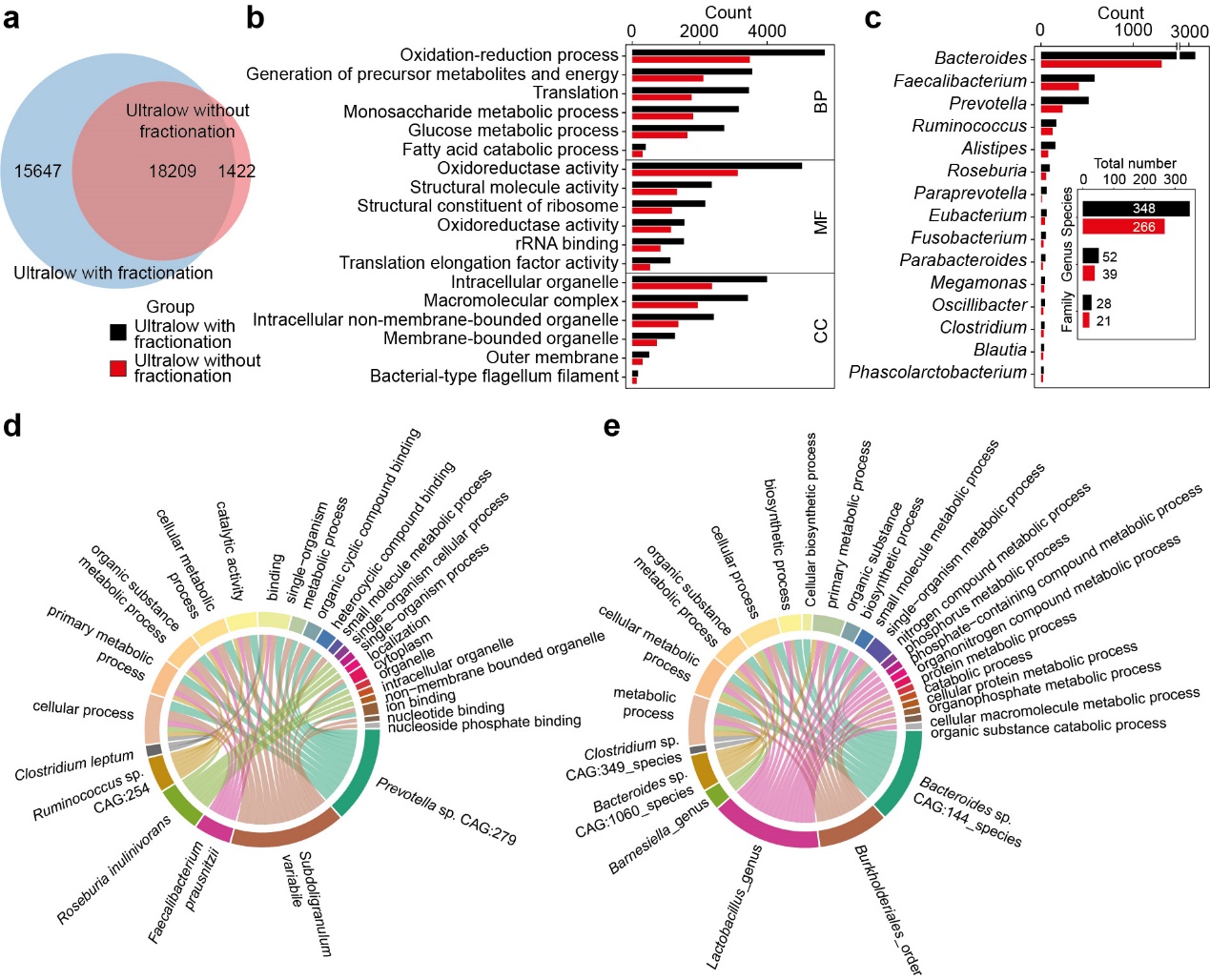


**Figure S3**. The performance of off-line fractionation metaproteomics. (a) Venn diagrams of protein groups identified in ultralow method with and without fractionations (three biological replicates). (b) Comparison of Gene Ontology (GO) enrichment based on proteins. The top 6 GO terms in biological process (BP), molecular function (MF) and cellular component (CC) are shown (n = 3). (c) The protein numbers identified in the top 15 bacteria genus and total taxa numbers at the family, genus and species levels (in the middle). (d) Bacteria at the species level and their main corresponding function by proteins only identified in the ultralow fractionation group. (e) Low abundance bacteria only identified in the ultralow fractionation group and their corresponding biological processes. The thickness of each node is proportional to the protein numbers of the corresponding functions and taxa (three biological replicates).


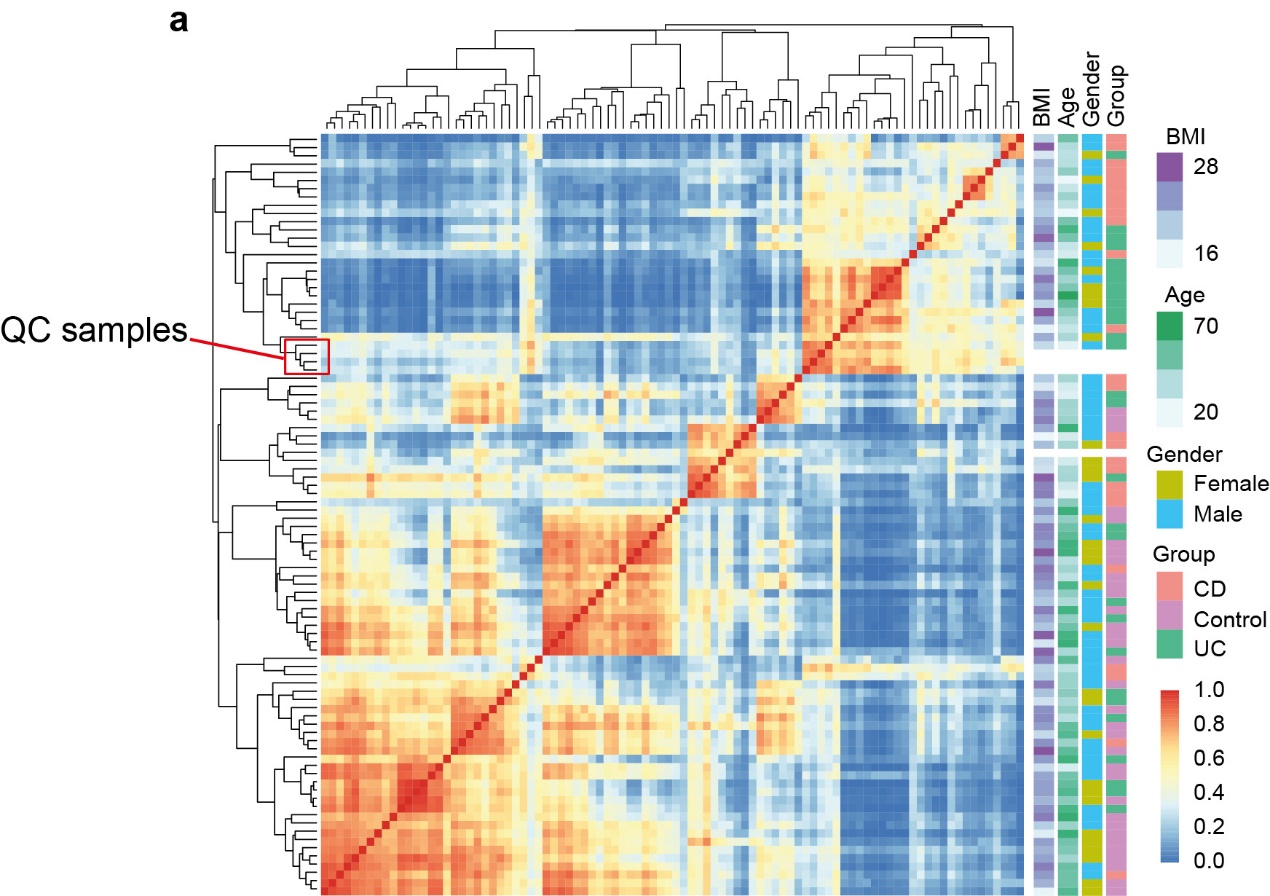


**Figure S4**. Sample-sample correlation heatmap calculated with identified metaproteomes across all samples (34 control samples, 26 CD samples, 29 UC samples, and 3 quality control (QC) samples) (a). Correlations are calculated with Pearson’s correlations and clustering with complete method both in row and column. Body mass index (BMI), age, gender, and disease group of all samples were shown.


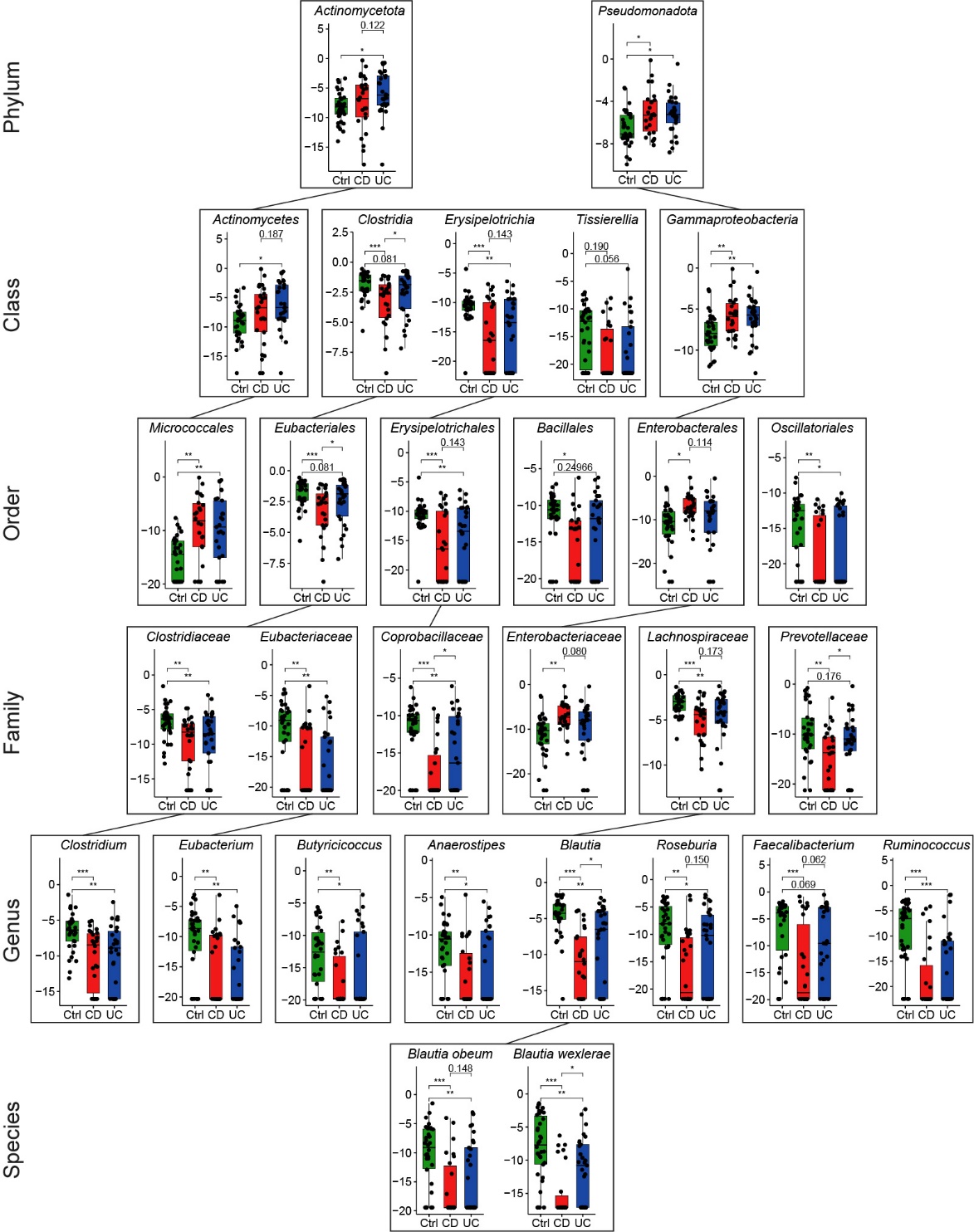


**Figure S5**. Taxonomical alterations in IBD patients. (a) Normalized relative abundance of altered microbiota at phylum, class, order, family, genus and species level (log_2_ transformed). Kruskal-Wallis test was employed to evaluate statistical difference among three groups (control, CD, and UC). Taxa with FDR < 0.05 were shown. The q value between two groups was calculated by MaAsLin2 to adjust age. For boxplots, all samples were shown with missing values replaced by a 0.2-fold minimum value. Box plots indicate the first (bottom line), medium (central line), and third (top line) quartiles of the data. **q* < 0.05, ***q* < 0.01, ****q* < 0.001.


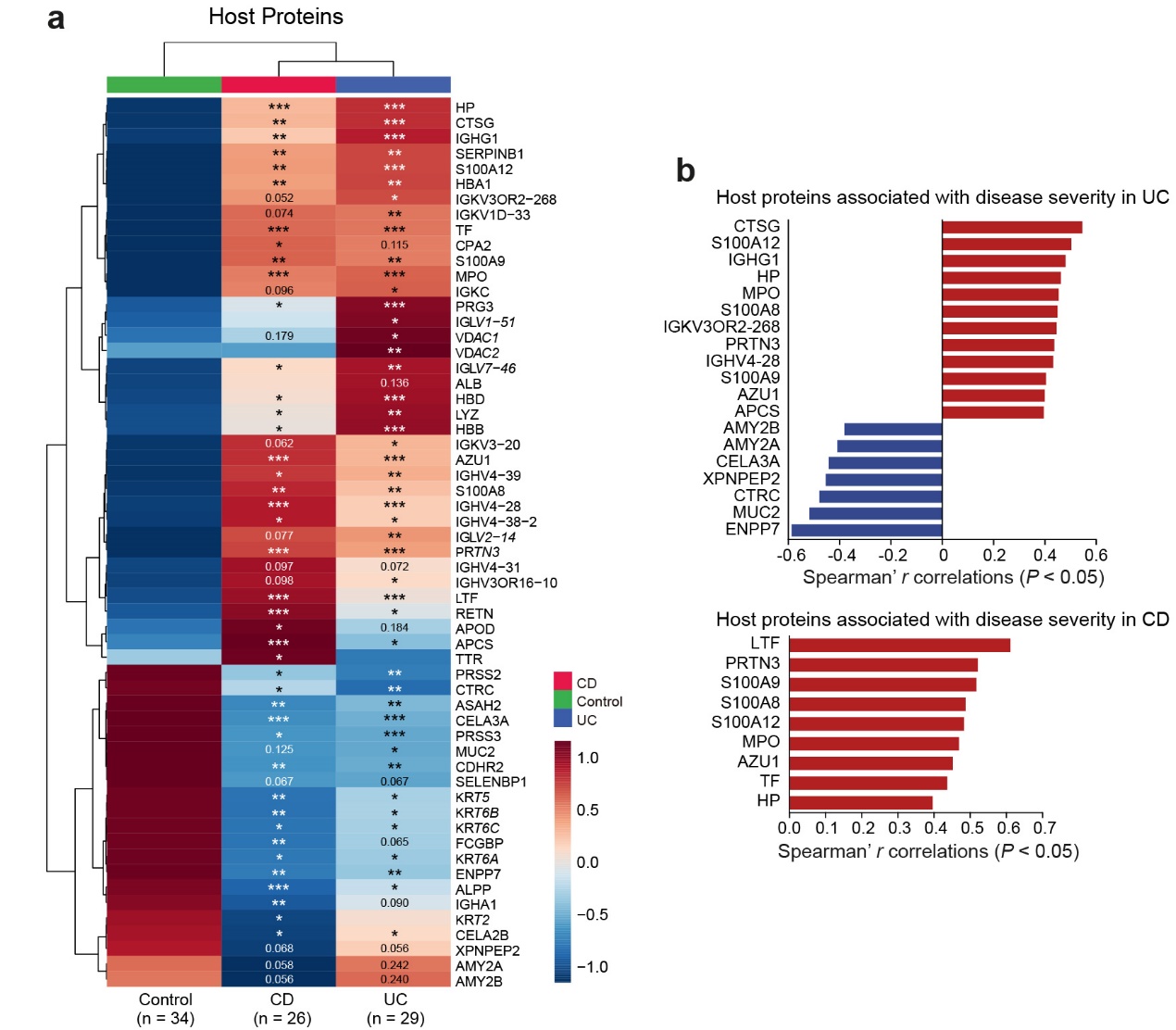


**Figure S6**. Alterations of host proteome in IBD patients. (a) Differential host proteins identified in IBD. Significant changes were evaluated by MaAsLin2 (*q* < 0.25) to adjust age between two groups after Kruskal-Wallis test (FDR < 0.05). The colors in the heatmap represent the average of relative abundance in each group. **q* < 0.05 versus control; ***q* < 0.01 versus control; ****q* < 0.001 versus control. (b) Correlations of disease severity and altered host proteome (Spearman’s rank correlation, *P* < 0.05). Red and blue colors indicate positive and negative correlations, respectively. CDAI scores for CD and UCAI scores for UC are used to determine disease severity. Only correlations with *P* < 0.05 are shown.


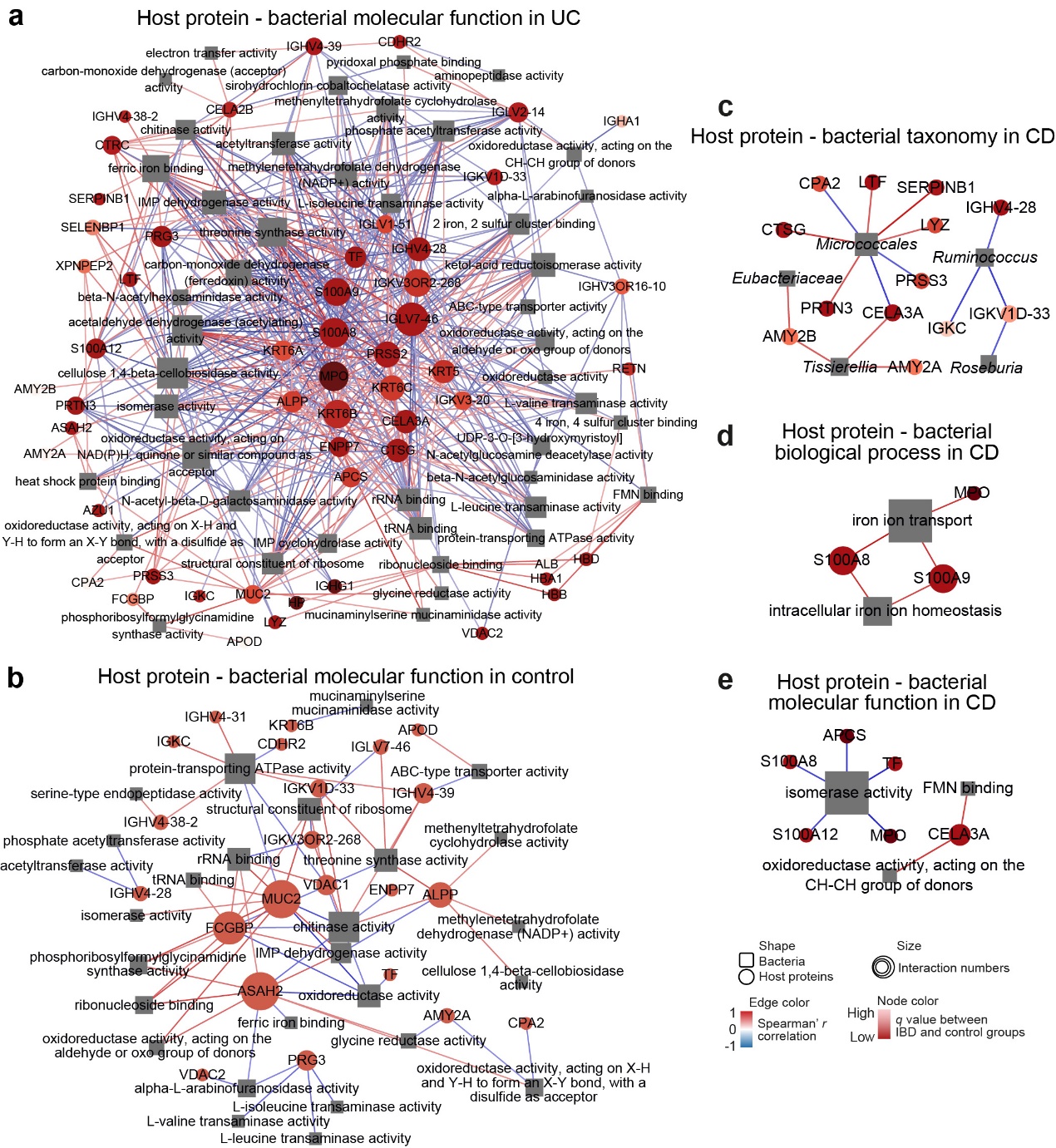


**Figure S7**. Co-occurrence networks of host proteins and microbiome that were differentially expressed in UC, CD, and control. Correlations of differential host proteins and altered microbial molecular functions in UC (a) and control (b) (FDR < 0.25). (c) Correlations of differential host proteins and altered gut microbial composition in CD (FDR < 0.25). (d) Correlations of differential host proteins and altered microbial biological processes in CD (FDR < 0.25). (e) Correlations of differential host proteins and altered microbial molecular functions in CD (FDR < 0.25). The correlations in networks were calculated by Spearman’s rank correlation (FDR < 0.25). The circle indicates human proteins, and the square indicates microbiome. The size and color of nodes are proportional to the connection number (degree) and *q* value, respectively. The host protein nodes in control are shown in the same color. The edge color is proportional to the Spearman’s rank correlation. Red and blue colors indicate positive and negative correlations, respectively.


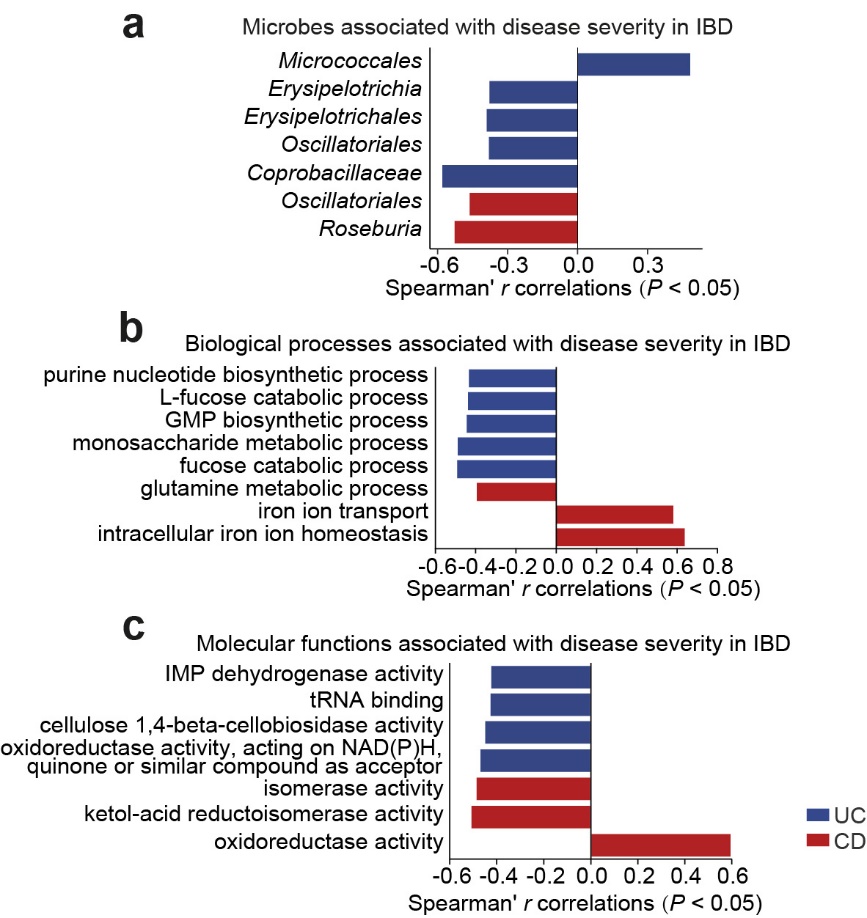


**Figure S8**. Correlations of disease severity and altered microorganisms (a), biological processes (b), and molecular functions (c) in UC and CD. The correlations in networks were calculated by Spearman’s rank correlation (*P* < 0.05). CDAI scores for CD and UCAI scores for UC are used to determine disease severity. Only correlations with *P* < 0.05 are shown.


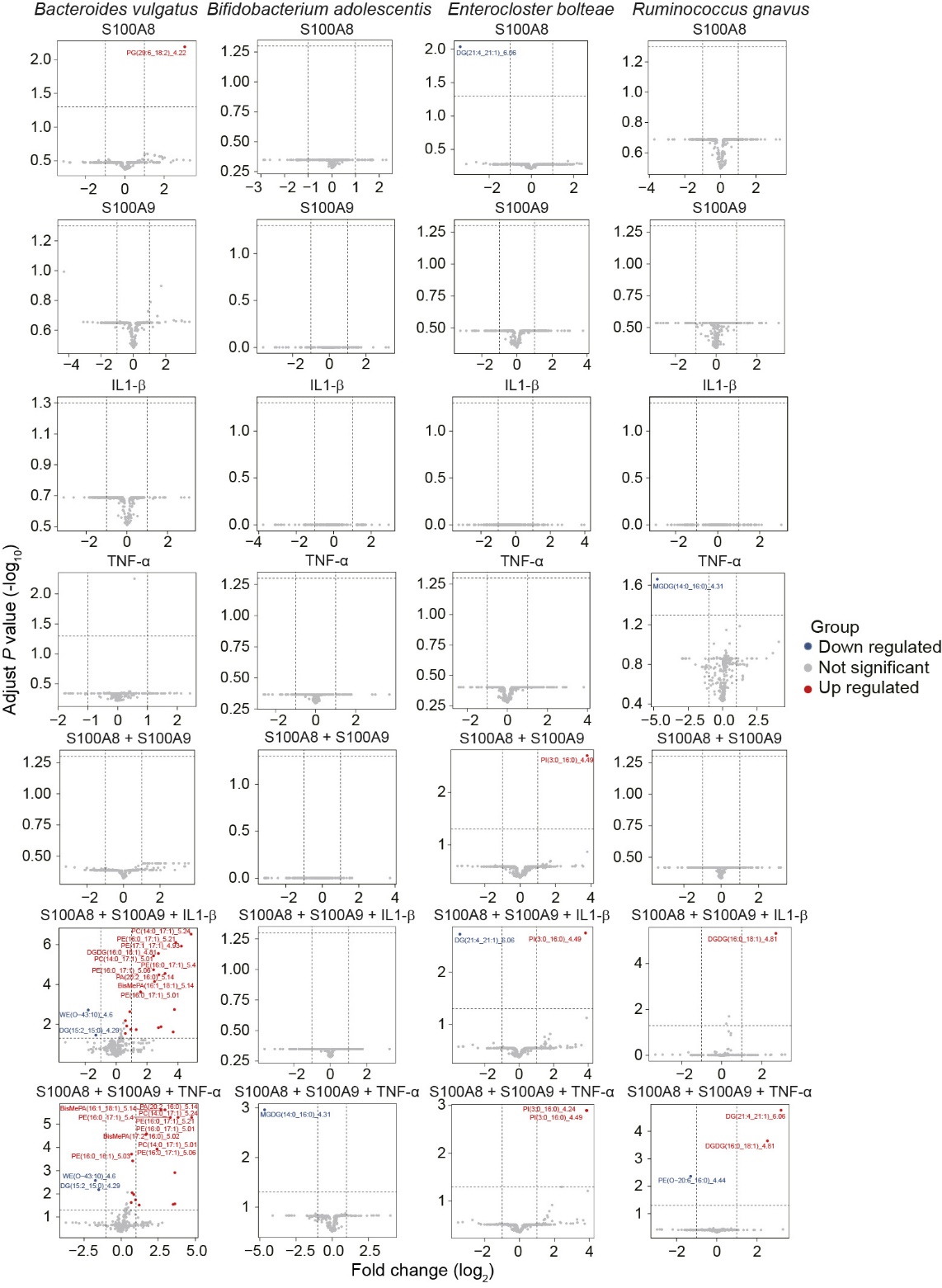


**Figure S9**. Bacterial lipidome alterations after inflammatory protein treatment in vitro. The volcano plots showed the discriminant lipids identified in inflammatory protein treatment group compared to control group (n = 3, adjusted *P* value < 0.05) in *Bacteroides vulgatus*, *Bifidobacterium adolescentis*, *Enterocloster bolteae*, *and Ruminococcus gnavus*.
